# Gut microbial composition and functionality of school-age Mexican population with metabolic syndrome and type-2 diabetes mellitus using shotgun metagenomic sequencing

**DOI:** 10.3389/fped.2023.1193832

**Published:** 2023-05-31

**Authors:** Ana K. Carrizales-Sánchez, Oscar Tamez-Rivera, Ricardo García-Gamboa, Tomás García-Cayuela, Nora A Rodríguez-Gutiérrez, Leticia Elizondo-Montemayor, Gerardo García-Rivas, Adriana Pacheco, Carmen Hernández-Brenes, Carolina Senés-Guerrero

**Affiliations:** ^1^ Tecnologico de Monterrey, Escuela de Ingenieria y Ciencias, Zapopan, Jalisco, Mexico; ^2^ Tecnologico de Monterrey, Escuela de Medicina y Ciencias de la Salud, Monterrey, Nuevo Leon, Mexico; ^3^ Tecnologico de Monterrey, Escuela de Medicina, Colonia Nuevo México, Zapopan, Jalisco, México; ^4^ Hospital Regional Materno Infantil de Alta Especialidad, Guadalupe, Nuevo Leon, Mexico; ^5^ Tecnologico de Monterrey, Institute for Obesity Research, Monterrey, Nuevo Leon, Mexico; ^6^ Tecnologico de Monterrey, Escuela de Ingenieria y Ciencias, Monterrey, Nuevo Leon, Mexico

**Keywords:** gut metagenome, metabolic syndrome, type-2 diabetes mellitus, school-age population, obesity

## Abstract

Gut metagenome in pediatric subjects with metabolic syndrome (MetS) and type-2 diabetes mellitus (T2DM) has been poorly studied, despite an alarming worldwide increase in the prevalence and incidence of obesity and MetS within this population. The objective of this study was to characterize the gut microbiome taxonomic composition of Mexican pediatric subjects with MetS and T2DM using shotgun metagenomics and analyze the potential relationship with metabolic changes and proinflammatory effects. Paired-end reads of fecal DNA samples were obtained through the Illumina HiSeq X Platform. Statistical analyses and correlational studies were conducted using gut microbiome data and metadata from all individuals. Gut microbial dysbiosis was observed in MetS and T2DM children compared to healthy subjects, which was characterized by an increase in facultative anaerobes (i.e., enteric and lactic acid bacteria) and a decrease in strict anaerobes (i.e., *Erysipelatoclostridium*, *Shaalia*, and *Actinomyces* genera). This may cause a loss of gut hypoxic environment, increased gut microbial nitrogen metabolism, and higher production of pathogen-associated molecular patterns. These metabolic changes may trigger the activation of proinflammatory activity and impair the host's intermediate metabolism, leading to a possible progression of the characteristic risk factors of MetS and T2DM, such as insulin resistance, dyslipidemia, and an increased abdominal circumference. Furthermore, specific viruses (*Jiaodavirus* genus and Inoviridae family) showed positive correlations with proinflammatory cytokines involved in these metabolic diseases. This study provides novel evidence for the characterization of MetS and T2DM pediatric subjects in which the whole gut microbial composition has been characterized. Additionally, it describes specific gut microorganisms with functional changes that may influence the onset of relevant health risk factors.

## Introduction

Metabolic syndrome (MetS) is a public health issue that may precede the onset of other relevant diseases, including type-2 diabetes mellitus (T2DM). MetS is characterized by the presence of at least three of the following cardiometabolic abnormalities: hypertension, increased central adiposity, hyperglycemia, hypertriglyceridemia, and decreased high-density lipoprotein (HDL) cholesterol (1). Sedentarism and unhealthy dietary patterns are strongly related to the progression of both diseases, as well as more adverse health outcomes compared to individuals under metabolic control (2).

In the last four decades, global childhood obesity has had a dramatic 10-fold increase among children and adolescents from five to 19 years of age, regardless of their socioeconomic status (3). It is known that a high body-mass index (BMI) in individuals aged seven years old to early adulthood increases the risk of developing adult T2DM if this condition is maintained until puberty or afterwards (4). Moreover, many pediatric subjects with obesity exhibit one or more metabolic disparities or cardiovascular risk factors that characterize MetS. It is estimated that MetS affects 6%–39% of children with obesity worldwide. This variation in the prevalence of MetS appears to be influenced by the criteria used to diagnose it, since there are no well-defined international diagnostic criteria for children and adolescents (5).

Currently, the physiological mechanisms that promote the progression of MetS are still unclear. However, it is thought that the gut microbiome is one possible factor that may affect the onset of this metabolic disease. Studies in toll-like receptor 5 (TLR5) deficient mice (T5KO) found a possible link between alterations in gut microbial communities and increased low-grade proinflammatory signaling that led to the development of MetS. Additional studies validated these findings with gut microbiota transplantation from T5KO into wild type (WT) germ-free mice (6, 7). Therefore, the study of gut microbial species and their role in human health has become increasingly relevant, placing the human microbiome as a potential therapeutic target for metabolic diseases. In this regard, some gut microbiome-based interventions (i.e., probiotics, prebiotics) have exhibited beneficial effects in treating MetS (8, 9).

Due to the limited knowledge about the role of the gut microbiota in the intermediary metabolism of pediatric subjects, this work focused on gut microbial taxonomic composition and functional potential in children and adolescents ages 7–17 years, stratifying them into three groups: healthy controls, MetS, and T2DM. Shotgun metagenomics was used to sequence stool samples and analyze correlations between the gut microbiome composition and functionality with impairments in the metabolism of glucose and fatty acids, increased proinflammatory activity, and potential loss of gut permeability.

## Material and methods

### Human study populations and collection of biological samples

**Figure 1:**
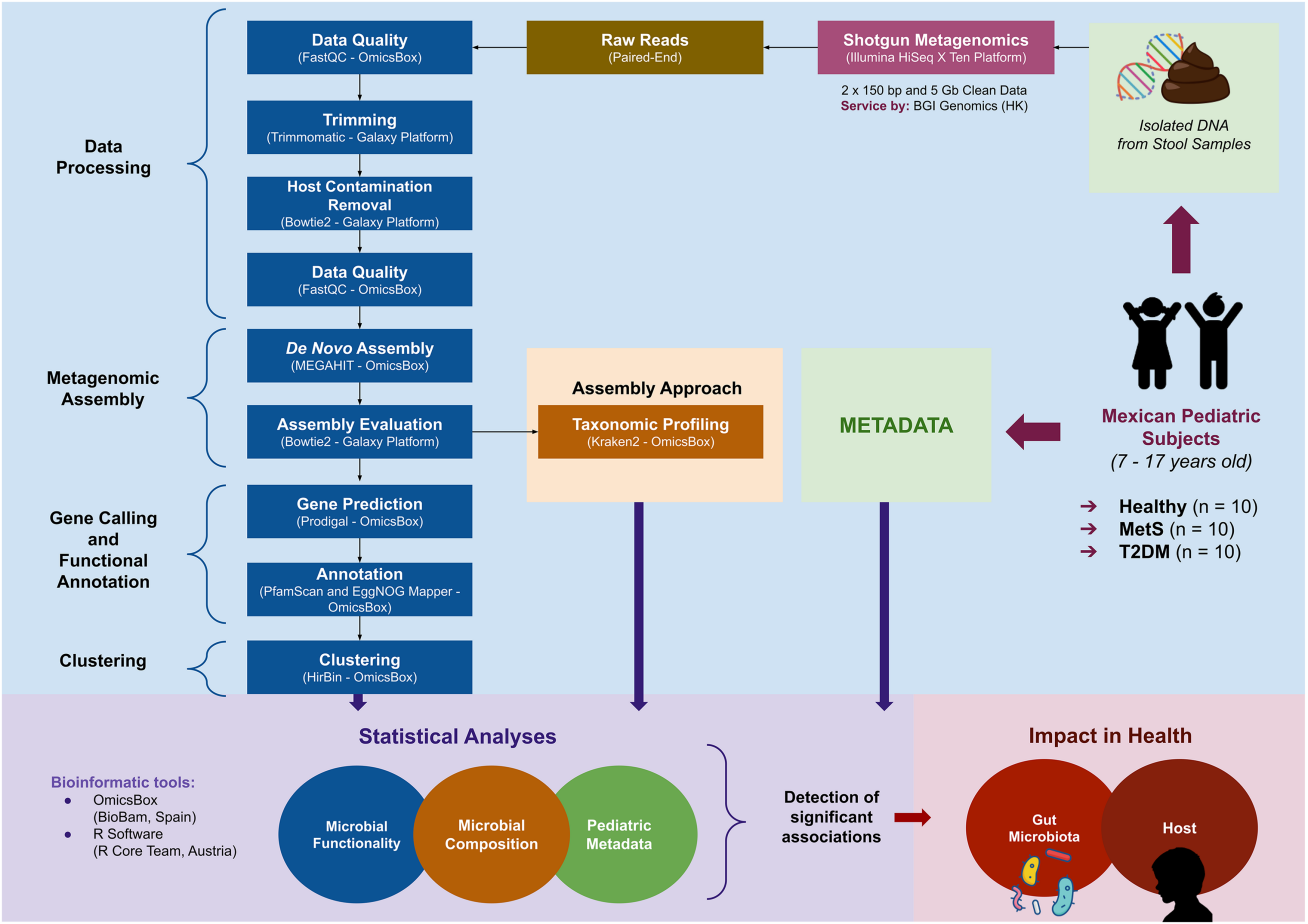
Workflow general overview of the approach used to study gut microbiota—host interactions in Mexican pediatric subjects: from an existent database, ten subjects were selected from each medical condition: healthy, metabolic syndrome (MetS), and type-2 diabetes mellitus (T2DM). Fecal DNA from the selected individuals was used to perform shotgun metagenomics. Metadata and information retrieved from the microbial composition and functionality of each study group were analyzed to detect significant associations between gut microbiota and each medical condition.

The general workflow described in Figure 1 was followed, where 30 subjects were selected from an internal database belonging to the “Cardiovascular and Metabolomics Medicine” research group from Tecnologico de Monterrey according to the best compliance with criteria for each condition: ten subjects considered as healthy, ten subjects with MetS, and ten subjects diagnosed with T2DM. Patients with MetS were chosen according to the modified criteria for adolescents proposed by Cook et al. (2003) (10). Subjects with T2DM were selected according to the American Diabetes Association diagnostic criteria (11). The control group included healthy subjects who were assessed by a pediatrician and had normal anthropometric measurements and biochemical parameters for their age and sex.

This database was previously created performing a non-probabilistic sampling for convenience at the *Hospital Regional Materno Infantil de Alta Especialidad*, located in Nuevo Leon, Mexico, where fecal and blood samples were obtained from 66 Mexican pediatric subjects after obtaining signed consents of their parents or legal tutors. Subjects with a positive history of antibiotic, probiotic and/or prebiotic use during the past three months were excluded, as well as subjects with underlying conditions that may modify their gut microbiota (i.e., inflammatory bowel disease, asthma, atopic dermatitis, active infection, hypo/hyperthyroidism). In addition to the biological samples, anthropometric measurements were obtained according to the National Health and Nutrition Examination Survey (NHANES) criteria.

### DNA extraction and metagenomic shotgun sequencing

Fecal samples were collected from each patient by members of the “Cardiovascular and Metabolomics Medicine” research group using the OMNIgene-GUT® OMR 200 kit to preserve samples at room temperature until DNA extraction. Total DNA was extracted from 500 mg of fecal samples using the FastDNA® Kit for Feces (MP Biomedicals, USA) according to the supplier's procedure, where a sample lysis step was previously done with a FastPrep® System Instrument (MP Biomedicals, USA). DNA was quantified using a NanodropTM Spectrophotometer (Thermo Fisher Scientific, USA) and stored at −80°C until analysis. DNA samples were sent to BGI Genomics (Hong Kong, CN) for shotgun metagenomic sequencing using the Illumina HiSeq X platform (Illumina Inc, California, USA), which generated paired-end 2 × 150 bp read length with ≥32 M reads and ≥5 Gb clean data per sample.

### Bioinformatic and statistical analyses

Data filtering of raw reads was conducted by the sequencing supplier, who removed adapter sequences and low-quality reads. For additional quality control, clean 150-bp paired-end reads were analyzed with FastQC (12) in the OmicsBox software (13). Then, Bowtie2 (14) was used to align sequence reads against the human genome assembly hg19 from UCSC Genome Browser (15) with default parameters to identify and remove potential human reads. Clean paired-end reads from each subject were individually subjected to metagenomic assembly using MEGAHIT (16) included in the OmicsBox software (13). Finally, Bowtie2 (14) was used to check the quality of the assemblies using the clean paired-end reads as a reference for this evaluation.

### Taxonomic classification and profiling of gut microbial functionality

Unless stated otherwise, all the bioinformatic tools applied in this module were executed using the OmicsBox software (13). Assemblies were compared with the non-redundant RefSeq database v.2020.07 (2020) using Kraken2 (17) to retrieve gut microbial composition with a confidence threshold score of 0 and ≥95.43% precision. To detect relevant functional potential, metagenomic gene prediction of assemblies was firstly conducted using Prodigal (18). Finally, its annotation was done with EggNOG Mapper (19). Alpha diversity at the species level of the study groups was analyzed by calculating Shannon Index (SI) with the Vegan package (20) available for R Studio software (2020) (21), and one-way ANOVA was conducted to determine if there was any difference between SI and medical conditions implicated in the study. In beta-diversity, Principal Coordinate Analysis (PCoA) applying Bray-Curtis distance was conducted at each taxonomic level among each study group, using the Vegan package (20). Finally, differential abundance analysis of microorganisms at each taxonomic level was done by conducting GLM Quasi Likelihood F-tests using the “Comparative analysis” function based on edgeR (22) included in the OmicsBox (13) metagenomic module. Differential abundance analysis of predicted genes that were successfully annotated from each individual in each study group was conducted using the “Comparative analysis” function in the OmicsBox (2019) (13) metagenomic module based on over-dispersed Poisson generalized linear modeling using the ortholog group descriptions (COG/KOG/NOG) and KEGG pathways. This module is based on the ShotgunFunctionalizeR library (23) and the HirBin tool (24). Metadata from the 30 selected patients was analyzed to detect significant associations with gut microbial composition and functional potential in each study group. Anthropometric, demographic, biochemical, and cytokine measurements were analyzed using one-way ANOVA for parametric variables and the Kruskal-Wallis test for nonparametric and ordinal variables. Significance was determined as *p* < 0.05, and interactions among study groups were analyzed using the Tukey HSD test, in the case of parametric data, and Dunn's test for nonparametric and ordinal data. Additionally, Fisher's exact test was used to study anthropometric and demographic variables with nominal binary attributes, where *p* < 0.05 was also used to determine significance. Finally, Pearson's correlation was used to detect possible significant associations between metadata and microbial taxonomic composition.

## Results

### Sequencing statistics

As shown in Supplementary Table S1, >394 M reads were retrieved from shotgun metagenomic sequencing of samples belonging to each study group: healthy, MetS, and T2DM. Of the analyzed reads, 0.06%–0.10% were removed since they were detected as possible contaminants belonging to the human genome. After read trimming, a taxonomic classification was obtained at >60% and a predicted gene annotation with EggNOG Mapper of >18%.

### Metadata statistics

The patients' metadata were analyzed, including demographic, clinical, and biochemical characteristics (Tables 1, 2). Mean age was higher in the T2DM and MetS groups compared to controls. A slight and non-statistically significant predominance of male subjects was observed in the control and T2DM groups. Nutritional assessment concluded that a western dietary pattern prevailed among the three groups; however, a comparative daily caloric intake analysis was not performed. As expected, subjects with MetS and T2DM exhibited statistically significant differences regarding body mass index (BMI), waist circumference (WC), and obesity compared to healthy subjects. Likewise, all patients with T2DM and 90% of those with MetS had hypertriglyceridemia (*p* < 0.001). Interestingly, subjects with T2DM had higher mean triglyceride levels compared to those with MetS (*p* < 0.001). High-density lipoprotein (HDL) cholesterol levels, known for their protective effect against cardiometabolic diseases, were significantly lower among children with MetS and T2DM. Indicators of insulin resistance (IR), such as fasting blood glucose (FBG) and serum insulin levels, were also significantly higher in the MetS and T2DM groups. This was further confirmed when comparing the homeostatic model assessment for IR (HOMA-IR) scores of patients with MetS and T2DM to those of healthy controls. As expected, patients with T2DM had significantly higher HOMA-IR scores than those with MetS (*p* < 0.001).

**Table 1:** Demographic, clinical and paraclinical data of children and adolescents with MetS, T2DM and healthy controls.

		Healthy (*n* = 10)	MetS (*n* = 10)	T2DM (*n* = 10)	Total
Sex	Male	6	5	6	17
	Female	4	5	4	13
	Total	10	10	10	30
	***p-value***	1			
Acanthosis nigricans	Yes	0	10	9	19
	No	10	0	1	11
	Total	10	10	10	30
	***p-value***	<0.001*^a,b^			
Overweight	Yes	0	3	2	5
	No	10	7	8	25
	Total	10	10	10	30
	***p-value***	0.321			
Obesity	Yes	0	6	7	13
	No	10	4	3	17
	Total	10	10	10	30
	***p-value***	<0.003* ^a,b^			
Hypertriglyceridemia	Yes	0	9	10	19
	No	10	1	0	11
	Total	10	10	10	30
	***p-value***	<0.001*^a,b^			
Low HDL	Yes	1	7	8	16
	No	9	3	2	14
	Total	10	10	10	30
	***p-value***	0.004*^a,b^			
High FBG	Yes	0	4	9	13
	No	10	6	1	17
	Total	10	10	10	30
	***p-value***	<0.001*^b^			
High CRP	Yes	3	NA	9	12
	No	7	NA	1	8
	Total	10	NA	10	20
	***p-value***	0.020*^b^			

Nominal binary variables were analyzed and determined according to the MetS Cook Criteria for adolescents (2003). Fisher's exact test was used for nominal binary variables. The significance among groups was established as a *p*-value <0.05*: ^a^represents the statistical difference between healthy and MetS, and ^b^between healthy and T2DM.

No differences were found between MetS and T2DM. Bold values represent statistical significance.

**Table 2:** Demographic, anthropometric, and biochemical measurements from healthy, MetS, and T2DM children and adolescents.

Parameter	Healthy (*n* = 10)	MetS (*n* = 10)	T2DM (*n* = 10)	*p*-value
Age	10.9 ± 2.3^b^	12.7 ± 1.8	14.4 ± 1.6^b^	0.002*
Height (cm)	144.8 ± 14.9^b^	157.0 ± 12.4	160.4 ± 7.6^b^	0.018*
Weight (kg)	38.13 ± 10.60^a,b^	70.22 ± 24.28^a^	80.23 ± 23.37^b^	<0.001*
BMI (kg/m^2^)	17.55 (16.60–17.95)^a,b^	25.30 (23.58–32.80)^a^	27.55 (26.13–34.48)^b^	<0.001*
BMI (%)	61.5 (43.8–67.3)^a,b^	97.5 (92.5–98.8)^a^	97.0 (93.5–98.8)^b^	<0.001*
WC (%)	25.0 (17.5–25.0)^a,b^	87.5 (75.0–93.8)^a^	82.5 (75.0–95.0)^b^	<0.001*
HC (cm)	66.2 (62.8–69.5)^a,b^	93.0 (88.8–111.8)^a^	97.5 (90.5–113.8)^b^	<0.001*
WHR	0.970 (0.963–0.980)	0.915 (0.840–0.960)	0.970 (0.953–0.980)	0.142
WHtR	0.445 (0.434–0.455)^a,b^	0.590 (0.517–0.635)^a^	0.586 (0.551–0.706)^b^	<0.001*
SBP (%)	39.5 (32.5–41.5)	73.5 (30.8–88.3)	63.0 (48.0–81.25)	0.058
DBP (%)	61.8 ± 9.8	62.9 ± 25.9	70.7 ± 20.2	0.555
TC (mg/dl)	142.0 (134.5–150.5)	174.5 (145.5–186.8)	147.0 (142.0–195.0)	0.164
TG (mg/dl)	79.7 ± 22.1^a,b^	152.7 ± 48.0^a^	204.7 ± 71.6^b^	<0.001*
HDL (mg/dl)	54.3 ± 15.5^a,b^	39.3 ± 9.2^a^	35.7 ± 7.3^b^	0.002*
LDL (mg/dl)	91.4 ± 21.7	94.4 ± 23.6	80.5 ± 20.1	0.339
FBG (mg/dl)	87.0 (83.5–89.0)^a,b^	95.5 (92.5–102.5)^a,c^	120.5 (111.8–151.0)^b,c^	<0.001*
Serum insulin (mIU/l)	6.40 (4.71–8.18)^a,b^	18.15 (15.25–30.18)^a^	25.95 (17.50–29.48)^b^	<0.001*
HOMA-IR	1.277 (0.971–1.773)^a,b^	4.515 (3.453–7.310)^a,c^	8.367 (6.133–9.872)^b,c^	<0.001*
Cook Criteria (0–5)	0 (0–0)^a,b^	3 (3–4)^a^	3.5 (3–4)^b^	<0.001*

Data are shown as mean ± SD for parametric data and median (Q25%–Q75%) for nonparametric data. Significance was established as a *p*-value ≤ 0.05*, where one-way ANOVA was used for parametric variables and the Kruskall-Wallis test for nonparametric variables. Interactions among study groups were analyzed using the Tukey HSD test for parametric data and Dunn's test for nonparametric data: ^a^represents the statistical difference between healthy and MetS, ^b^between healthy and T2DM, and ^c^between MetS and T2DM.

Metabolic diseases are considered proinflammatory conditions. For this reason, we compared C-reactive protein (CRP) levels in patients with T2DM and healthy controls. Interestingly, the vast majority (90%) of the subjects with T2DM had high levels of CRP (>0.9 mg/dl), compared to only 30% of the control group (*p* = 0.020). In the case of cytokine measurements (Table 3), only monocyte chemoattractant protein-1 (MCP-1), IL-8, and interleukin (IL)-18 were significantly higher between T2DM and healthy pediatric subjects without detecting a significant difference in the MetS group.

**Table 3:** Cytokine profile of children and adolescents with MetS, T2DM, and healthy controls.

Cytokine (pg / mL)	Healthy (*n* = 10)	MetS (*n* = 10)	T2DM (*n* = 10)	*p*-value
IL-1β	0.635 (0.130–1.665)	0.550 (0.355–1.070)	0.855 (0.590–2.588)	0.414
IFN-α2	0.130 (0.110–3.763)	3.285 (0.203–12.245)	0.265 (0.165–1.265)	0.413
IFN-γ	1.125 (0.250–3.285)	3.090 (1.258–4.258)	4.315 (1.965–5.380)	0.096
TNF-α	1.548 ± 1.600	1.558 ± 0.890	1.285 ± 0.703	0.830
MCP-1	199.355 ± 46.178^b^	220.861 ± 65.885	274.966 ± 79.447^b^	0.042*
IL-6	2.841 ± 1.738	3.637 ± 1.726	3.316 ± 1.474	0.562
IL-8	4.718 ± 2.836^b^	5.782 ± 3.973	9.961 ± 5.953^b^	0.033*
IL-10	1.416 ± 1.055	1.758 ± 0.839	2.236 ± 1.196	0.227
IL-12p70	0.310 (0.120–0.698)	0.505 (0.230–0.883)	0.625 (0.313–0.785)	0.592
IL-17A	6.700 (1.500–12.843)	6.475 (4.915–18.138)	6.135 (4.548–11.878)	0.619
IL-18	118.465 (72.810–167.515)^b^	150.575 (99.140–221.350)	204.390 (180.033–262.700)^b^	0.011*
IL-23	4.855 (2.018–6.693)	3.710 (2.985–7.273)	5.940 (2.133–7.040)	0.904
IL-33	4.900 (0.740–11.000)	6.425 (4.138–16.563)	12.975 (5.825–15.050)	0.407

Data are shown as mean ± SD for parametric data and median (Q25%–Q75%) for nonparametric data. Significance was established as a *p*-value ≤ 0.05*, where one-way ANOVA was used for parametric variables and the Kruskall–Wallis test for nonparametric variables. Interactions among study groups were analyzed using the Tukey HSD test for parametric data and Dunn's test for nonparametric data: ^b^represents the statistical difference between healthy and T2DM. No statistical differences between healthy and MetS and MetS and T2DM were found.

### Microbial taxonomic analysis in healthy, MetS, and T2DM study groups

Taxonomic results showed that the Bacteria domain (>97%) was the most abundant in all study groups, followed by Eukarya (∼2.25%), Archaea (∼0.38%), and virus (∼0.13%). Statistical analyses showed no significant differences between the abundance of each microbial domain and the studied groups (Supplementary Figure S1). However, a trend was perceived for the Bacteria and Eukarya domains: Bacteria seemed to increase while Eukarya decreased from a healthy state to a T2DM condition. No visible trend was detected with viruses among the study groups.

**Figure 2:**
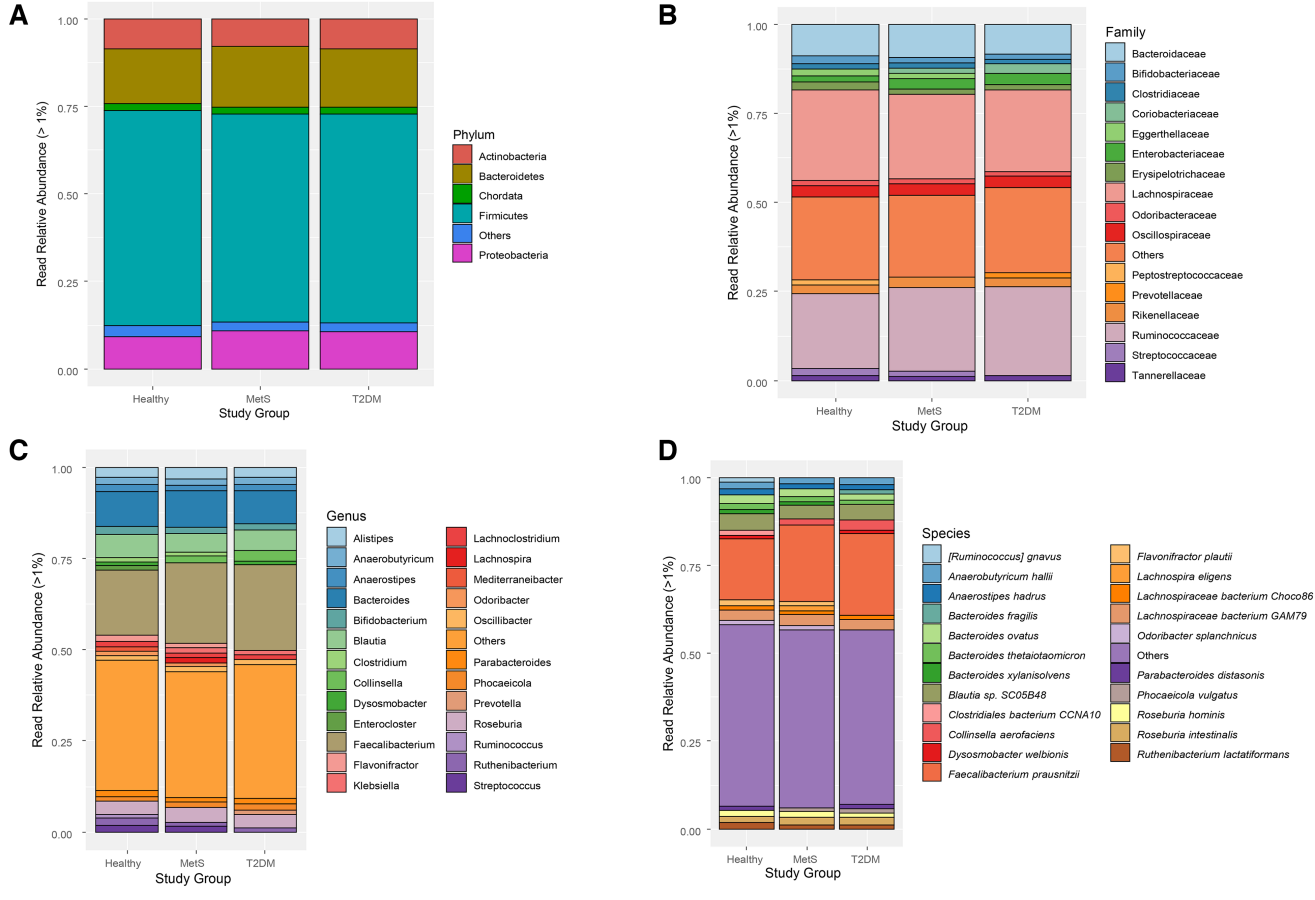
Taxonomic classification of gut microbiota in each study group: (**A**) phylum, (**B**) family, (**C**) genus, (**D**) Species. “Others” groups: microbial species with less than 1% of relative read abundance.

Read relative abundance was calculated for phylum, family, genus, and species levels. There were no evident changes between the study groups at phylum and family levels (Figures 2A,B), but changes started to be visible at genus and species levels (Figures 2C,D). Firmicutes and Bacteroidetes were the most abundant phyla within the three study groups, detecting a slight increase in Bacteroidetes and a slight decrease in Firmicutes within the MetS and T2DM groups compared to the healthy individuals. The relative abundance of Proteobacteria slightly increased in the MetS and T2DM groups (Figure 2A).

Moreover, Ruminococcaceae and Lachnospiraceae were the most abundant families in the gut microbiome of these pediatric subjects (Figure 2B). Streptococcaceae, Peptostreptococcaceae, Lachnospiraceae, Eggertellaceae, Erysipelotrichaceae, and Bifidobacteriaceae families showed a decreasing trend in the MetS and T2DM conditions; whereas Prevotellaceae, Ruminoccocaceae, Enterobacteriaceae, and Coriobacteriaceae increased in these two conditions. At the genus level (Figure 2C), a depletion of *Streptococcus*, *Ruthenibacterium*, *Ruminococcus*, *Odoribacter*, *Mediterraneibacter, Lachnoclostridium*, *Klebsiella*, *Flavonifractor*, and *Enterocloster* was observed in the MetS and T2DM groups compared to the healthy condition, which could be an indicator of loss in gut microbial diversity. Notably, an increase in the relative read abundance of *Prevotella* and a decrease in *Bifidobacterium* was also observed in T2DM individuals compared to healthy subjects.

Finally, at the species level (Figure 2D), there was a decrease in *Ruminococcus gnavus*, *Clostridiales bacterium* CCNA10, *Flavonifractor plautii*, and *Ruthenibacterium lactatiformans* in subjects with MetS and T2DM compared to healthy conditions. Subjects with MetS showed decreased relative read abundance of *Parabacteroides diastasonis* and *Dysosmobacter welbionis*. On the other hand, an increase in *Bacteroides fragilis* and *Faecalibacterium prausnitzii* were perceived from a healthy state to a T2DM condition.

The Firmicutes/Bacteroidetes (F/B) ratio and the alpha diversity of the subjects' gut microbiota at the species level were analyzed (Supplementary Table S2, Figure S2); however, no statistically significant differences were observed among the three study groups.

**Figure 3:**
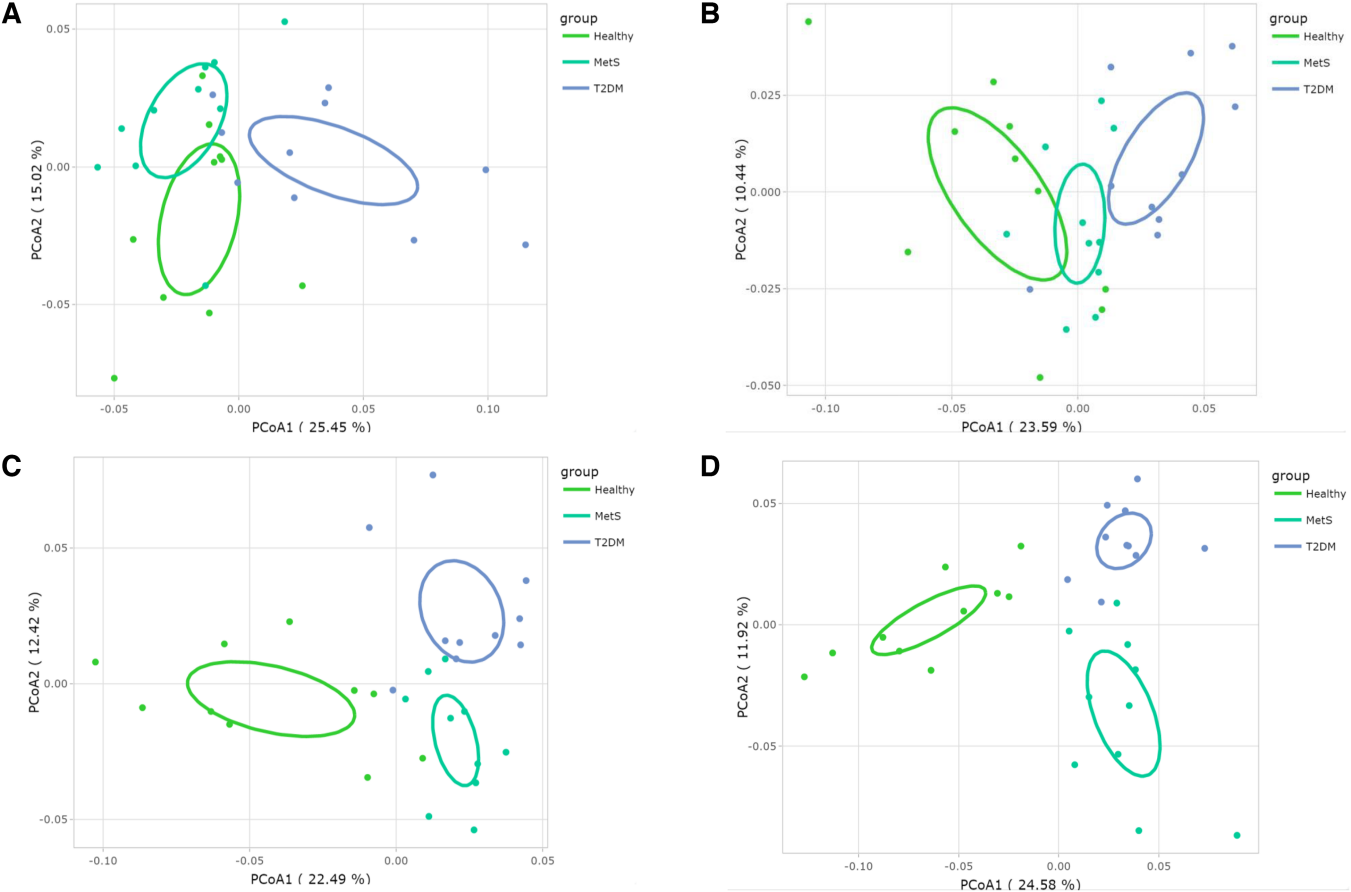
Gut microbiota's beta-diversity at different microbial taxonomy levels in Mexican children and adolescents: PcoA plots based on Bray-Curtis distance matrix (**A**) phylum, (**B**) family, (**C**) genus, and (**D**) species.

Prior to analyzing the beta-diversity at the species level, one-way ANOVAs were conducted to detect which microorganisms were significantly relevant among the studied groups. Afterwards, PCoAs were constructed considering the significant communities (Figure 3), showing clear differences at genus and species levels with 95% confidence.

**Figure 4:**
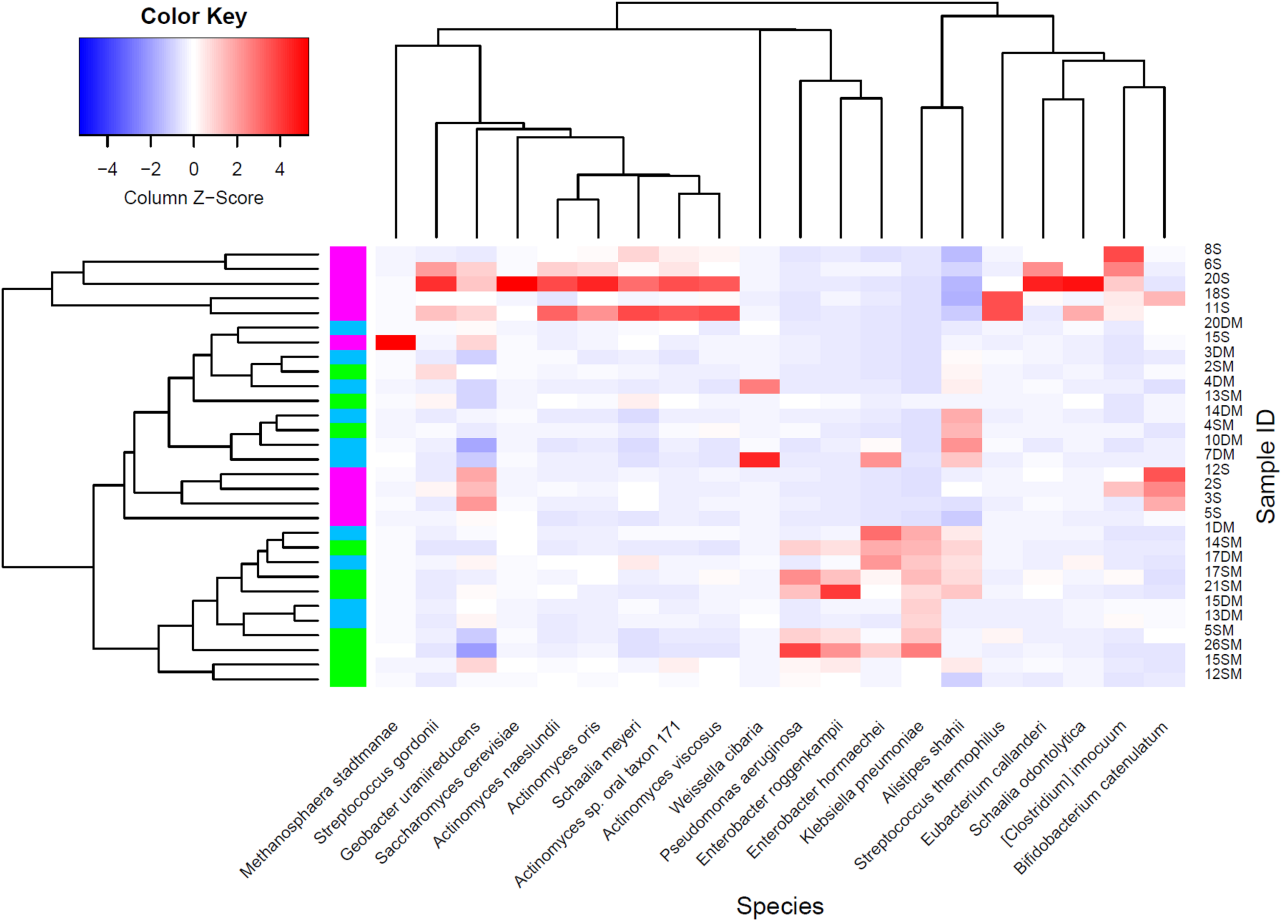
Statistically significant microorganisms at species level comparing MetS and T2DM with healthy individuals as a control group (MetS + T2DM vs. healthy): healthy subjects (magenta), MetS (green), and T2DM individuals (blue).

Since one-way ANOVA analyses at each taxonomic level showed a broad list of significant microorganisms, empirical Bayes Quasi Likelihood F-Tests were additionally performed to consider possible uncertainties in the estimation of abundance dispersion and a more reliable error rate control due to the limited sample size used per study group. Significant microorganisms detected at order, family, and genus levels are enlisted in Table 4, while Figure 4 and Supplementary Figure S3 show the statistically significant microorganisms at the species level.

**Table 4:** Statistically significant microorganisms at order, family, and genus levels comparing study groups.

Study groups	Taxonomic level	Scientific name	logFC	*p*-value	FDR
MetS and T2DM vs. healthy	Order	Actinomycetales	−1.775	<0.001	0.012
	Family	Actinomycetaceae	−1.769	<0.001	0.033
	Genus	*Erysipelatoclostridium*	−2.087	<0.001	0.004
		*Schaalia*	−2.758	<0.001	0.016
T2DM vs. healthy	Family	Leuconostocaceae	3.109	<0.001	0.033
		Lactobacillaceae	1.272	<0.001	0.042
		Rudiviridae	−3.251	<0.001	0.042
	Genus	*Weissella*	3.693	<0.001	0.010
		*Jiaodavirus*	5.546	<0.001	0.016
		*Pediococcus*	2.961	<0.001	0.047
		*Erysipelatoclostridium*	−2.035	<0.001	0.047
		*Acidaminococcus*	2.528	<0.001	0.047
T2DM vs. MetS	Genus	*Jiaodavirus*	6.531	<0.001	0.007
		*Weissella*	3.754	<0.001	0.007
		*Acidaminococcus*	2.941	<0.001	0.013
		*Kosakonia*	3.104	<0.001	0.042
		*Megamonas*	−3.403	<0.001	0.042
		*Pediococcus*	2.914	<0.001	0.042

GLM Quasi Likelihood F-Test was used for the statistical analysis. logFC, logarithm of fold change (FC), which is the ratio between the mean abundance value of each microorganism's OTUs in the contrast condition and the same value in the reference condition. logFC > 1 is defined as overrepresented, logFC < −1 as underrepresented, and significance was established as *p*-value ≤ 0.05. False discovery rate (FDR) was used as a corrected *p*-value where <0.05 was considered to determine differential abundance.

*Erysipelatoclostridium* and *Schaalia* were significantly less abundant in MetS and T2DM individuals compared to the healthy group. At the family level, Leuconostocaceae and Lactobaciliaceae were statistically higher in the T2DM group compared to the healthy one, contrary to Rudiviridae, which was significantly lower. *Jiaodavirus, Pediococcus, Weissella*, and *Acidaminococcus* resulted significantly higher in T2DM individuals than in healthy and MetS individuals. Finally, *Kosakonia* was significantly higher in T2DM individuals compared to the MetS group, and *Megamonas* was significantly lower (Table 4).

At the species level, most of the detected significant microorganisms belonged to the Actinobacteria, Firmicutes, Bacteroidetes, and Proteobacteria phyla. Specific members from Actinobacteria (i.e., *Actinomyces*, *Schaalia,* and *Bifidobacterium* spp.) were lower; while certain members from Proteobacteria (i.e., *Klebsiella*, *Kosakonia*, *Citrobacter*, *Proteus*, and *Enterobacter* spp.) were higher in MetS and T2DM groups, respectively. *Clostridioides innocuum* and *Geobacter uraniireducens*, which respectively belong to the Firmicutes and Proteobacteria phyla, were significantly higher in the healthy group compared to the others. Finally, *Pseudomonas aeruginosa* was significantly higher in MetS individuals when compared to the healthy and T2DM study groups.

### Microbial functional potential in healthy, MetS, and T2DM individuals

The obtained contigs were also used for gene prediction and annotation to study gut microbial functional potential. Genes were grouped according to their predicted functionality in the following categories: Information Storage and Processing, Cellular Processes and Signaling, and Metabolism. There were no significant statistical differences among study groups in any of the categories (Supplementary Figure S4). However, an increasing trend was observed in the “Metabolism” category from a healthy state towards the T2DM group (Supplementary Figure S4C). Sub-categories were also analyzed (Supplementary Figure S5) and no significant differences were observed among the studied groups.

Further data analyses included differential abundance analyses of KEGG pathways and ortholog group descriptions (COG/KOG/NOG) to identify the most significantly overrepresented and underrepresented pathways and genes in each study group. (Supplementary Tables S3, S4). Results from KEGG pathways showed that the nitrogen metabolism pathway was significantly overrepresented in MetS and T2DM individuals compared to the healthy group (FDR = 0.006; Supplementary Table S5), and also showed to be overrepresented in the T2DM condition compared to the MetS study group (FDR = 0.011; Supplementary Table S6).

Regarding significant overrepresented and underrepresented genes in MetS and T2DM subjects (Supplementary Tables S3, S4), genes belonging to relevant metabolic routes such as oxidative phosphorylation, flagellum and biofilm formation, fatty acid biosynthesis, and nitrification pathway were significantly overrepresented. On the other hand, genes belonging to cAMP-dependent protein kinase, Ca^2+^ homeostasis, citrate cycle, riboflavin, methane and tryptophan metabolisms and branched-chain amino acids (BCAA) degradation were shown to be underrepresented.

### Significant correlations between anthropometric data and microbial taxonomic composition

**Figure 5:**
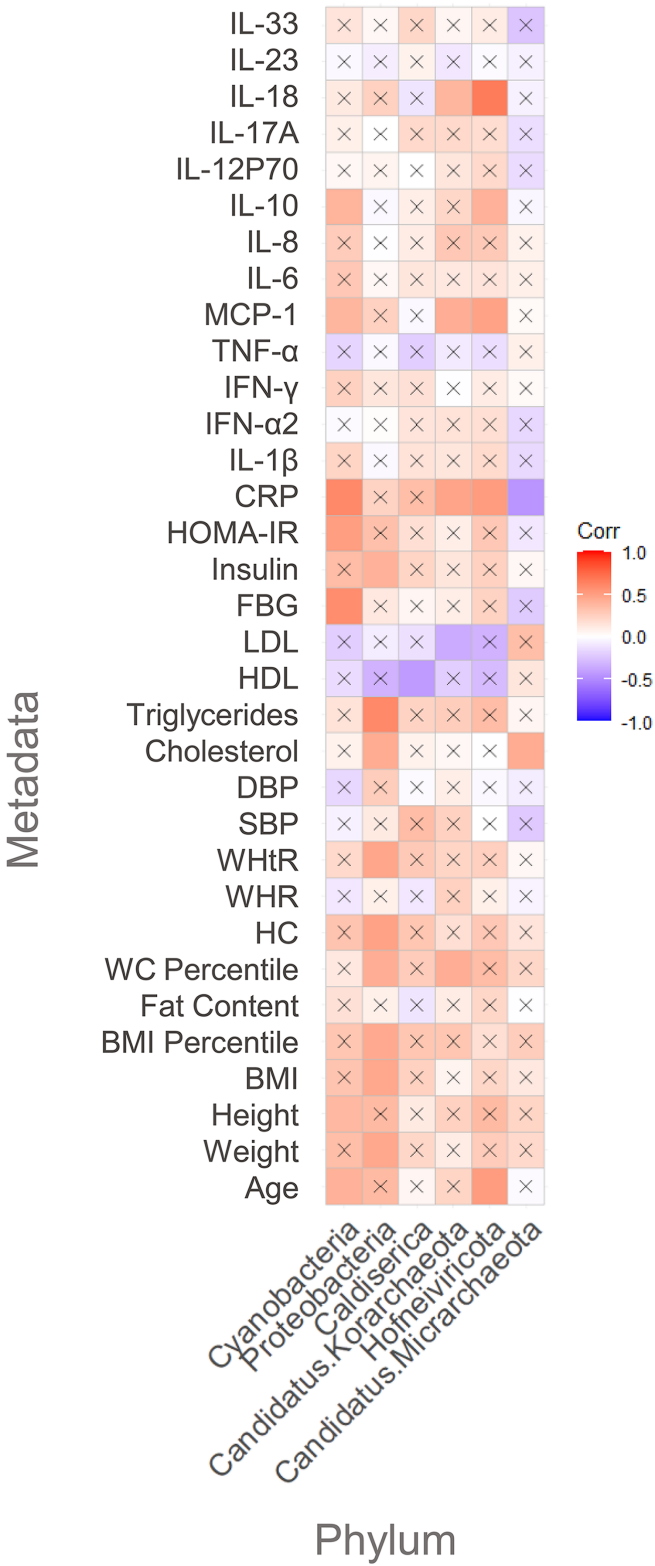
Pearson's correlation between metadata and significant phyla: “X” represents no significant correlation between the analyzed host's parameter and the analyzed phylum; the red color scale indicates a positive correlation, and the purple color scale indicates a negative correlation between the studied combination.

At the phylum level (Figure 5), Proteobacteria were positively and significantly correlated with relevant anthropometric factors related to MetS risk factors including: BMI percentile, TG levels, and insulin levels. Total cholesterol (TC) levels, weight, waist-to-height ratio (WHtR), and WC percentile were also positively correlated with this phylum. Interestingly, Cyanobacteria were positively correlated with FBG levels and HOMA-IR, which are indicators of impaired glucose metabolism in humans. Regarding cytokine profile, MCP-1, IL-10, and CRP also seemed to be positively correlated with Proteobacteria.

Other interesting findings at the phylum level were the significantly negative correlations between Caldiserica and HDL levels and Candidatus Korarchaeota with low density lipoprotein (LDL) levels. Hofneiviricota was positively correlated with MCP-1, IL-10, and IL-18. Finally, CRP showed to be positively correlated with Hofneiviricota and negatively correlated with Candidatus Micrarchaeota, belonging to the virus and Archaea domain, respectively.

At the family taxonomic level (Supplementary Figure S6), Inoviridae showed a positive correlation with MCP-1, IL-10, IL-18, and CRP levels. Retroviridae and Rudiviridae families were negatively correlated with IL-8 and MCP-1, respectively, and both showed a negative correlation with HDL levels. Likewise, Retroviridae was negatively correlated with HOMA-IR, insulin levels, and CRP levels. In regard to bacterial communities, Lactobacilliaceae was positively correlated with MCP-1, CRP, TG, head circumference (HC), and WC percentile. Desulfobacteriacea was negatively correlated with characteristic features of IR (HOMA-IR and insulin levels), increased abdominal obesity (weight, BMI, WC percentile, and HC), hypertension (SBP percentile), and dyslipidemia (HDL levels).

At the genus level (Supplementary Figure S7), *Klebsiella* spp. had a positive correlation with known risk factors for MetS, including impaired glucose (HOMA-IR and insulin levels) and increased abdominal circumference (weight, BMI percentile, WC percentile, and HC). *Weissella* and *Lactobacillus* spp. showed a positive correlation with weight, BMI, HOMA-IR, and CRP levels. Moreover, *Weisella* spp. also showed a strong positive correlation with FBG levels. Viral communities also showed interesting correlations. *Jiaodavirus* was positively correlated with proinflammatory activity (IL-18, IL-10, MCP-1), IR (HOMA-IR and insulin levels), and hypertension (DBP percentile). *Rudivirus* was positively correlated with HDL levels and negatively correlated with MCP-1. *Erysipelatoclostridium* was negatively correlated with increased abdominal circumference metadata (BMI percentile and WC percentile), TG levels, and CRP. *Acidaminococcus* spp. was positively correlated with TG and TC levels. *Actinomyces* spp. was negatively correlated with parameters related to increased abdominal circumference and positively correlated with HDL levels. Finally, *Desulfobacterium*, *Desulfitobacterium,* and *Pseudodesulfovibrio* spp. were negatively correlated with relevant metadata for almost all MetS risk factors, except those related to hypertension. Additionally, *Pseudodesulfovibirio* spp. showed a positive correlation with HDL levels.

At species level (Supplementary Figure S8), enterobacterial communities (i.e., *Klebsiella pneumoniae*, *Klebsiella oxytoca*, *Enterobacter* sp. HK169, *Proteus mirabilis*, *Enterobacter homaechei*, and *Enterobacter cloacae*) were positively correlated with metadata related with almost all MetS risk factors, except with those related to hypertension. Lactic acid bacteria (i.e., *Lactobacillus coryniformis*, *Lactobacillus ruminis*, *Weissella confusa*, and *Weissella cibaria*) were positively correlated with metadata associated with IR, increased abdominal circumference, dyslipidemia, and inflammation (CRP levels). In contrast, *Lactobacillus malefermentans* showed a negative correlation with TG and positive correlation with HDL levels. *Alistipes shahii* was positively correlated with increased abdominal circumference, dyslipidemia (TG levels), IR, and inflammation (CRP), and it also showed a negative correlation with LDL and HDL levels. Furthermore, microbial communities belonging to the Actinobacteria phylum (i.e., *Schaalia meyeri*, *Actinomyces viscosus*, and *Bifidobacterium pseudocatenulatum*) showed a negative correlation with increased abdominal circumference, IR (FBG levels), and dyslipidemia risk factors. Additionally, *Actinomyces viscosus* and *Bifidobacterium pseudocatenulatum* were positively correlated with HDL levels. Lastly, *Schaalia meyeri* was negatively correlated with MCP-1 and positively correlated with HDL.

## Discussion

### MetS and T2DM study groups possess more than three metabolic changes

MetS and T2DM study groups had more than three risk factors for MetS described by Cook et al. (10), including increased abdominal circumference (WC percentile), dyslipidemia (low HDL and high TG levels), and IR (high serum insulin levels, high FBG levels, and increased HOMA-IR) (Tables 1, 2). Clinical data also agreed with previous studies, which have significantly and positively associated WC with body fat content percentage (25). Both obese and overweight individuals were present in the MetS and T2DM groups. Obesity was slightly more prevalent in the T2DM group. Regarding TG levels, it has been reported that fluctuation may occur due to exogenous (i.e., dietary) and endogenous sources (i.e., hepatic metabolism). This is relevant to human metabolism due to their contribution to energy production, fat storage, lipolysis, and very low-density lipoprotein (VLDL) synthesis through the re-esterification of free fatty acids (FFA) in the liver (26). On the other hand, HDL is crucial in cholesterol disposal conducted by the liver when it is excessively present in peripheral blood, protecting against oxidative damage mediated by LDL, and conferring antiatherogenic and anti-inflammatory properties (27). Notably, cholesterol and LDL levels in the present study were not significantly different among groups; however, significantly decreased HDL levels within MetS and T2DM individuals may demonstrate a possible loss of cardioprotective properties and activation of inflammatory responses.

Interestingly, in the case of IR, serum insulin levels were statistically higher in MetS and T2DM compared to the healthy group. Still, FBG levels showed to be statistically different among the three study groups, indicating a significant gradual loss of glucose tolerance as the progression of the disease (T2DM) took place. This observation was confirmed by the HOMA-IR parameter, since FBG and serum insulin levels were used for its calculation; values were then significantly different among the three groups and gradually increased from a healthy to a T2DM condition (28). SBP and DBP did not show statistical differences between the studied groups, suggesting that based on those clinical measurements the individuals in the study did not exhibit hypertension-related trends.

### Increased levels of cytokines were significantly detected in pediatric individuals with T2DM

T2DM individuals presented significantly higher CRP levels compared to the healthy group. Unfortunately, CRP levels were not available in the MetS group. CRP is an acute inflammatory protein that increases in the presence of injury, inflammation, or infection. It is mainly produced by hepatocytes, but it can also be produced by other cells belonging to smooth muscle, endothelium, macrophages, adipocytes, and lymphocytes (29). Recently CRP levels have been positively correlated with dyslipidemia, MetS, and T2DM in Korean (30) and American adults (31). Notably, CRP production has also been positively correlated with increased proinflammatory cytokines such as IL-6, IL-8, MCP-1, and tumor necrosis factor (TNF)-*α* (29). In this study, MCP-1 and IL-8 were significantly different in T2DM pediatric subjects compared to healthy individuals, possibly explaining the significant increase in CRP levels within the diabetic group (Table 3).

In humans, IL-8 may be produced by multiple cells, particularly by the intestinal epithelial cells (32). Angrisano et al. (33) observed that the activation of IL-8 in intestinal epithelial cells may be triggered by bacterial lipopolysaccharides (LPS). Additionally, IL-8 is considered one of the most effective neutrophil chemoattractants, recruiting neutrophils into the epithelial barrier (32). Neutrophils are antimicrobial effector producers by degranulation processes (29) and are involved in the loss of junction proteins, increased epithelial permeability, and intestinal inflammation (34).

As previously stated, IL-18 was significantly higher in T2DM individuals compared to the healthy group. High IL-18 levels have been associated with interferon (IFN)-γ induction. Previous studies have concluded that IFN-γ is involved in the pathogenesis of T2DM, and is associated with known MetS risk factors such as obesity, IR, dyslipidemia, and hypertension (35). In the present study, there were no significant differences in IFN-γ levels between the studied groups; however, an increasing trend was observed from a healthy to a T2DM state. Pahwa et al. (37) reported that the production of IL-18 may be triggered by an increased activity of NLRP3 inflammasome from the innate immune system in subcutaneous adipose tissue, which is formed because of increased activation of the NOD-like receptor signaling pathway (36).

Previous studies have reported the relevance of MCP-1 in recruiting macrophages and monocytes to inflamed tissues, not only for bacterial recognition and elimination, but also to achieve immune system homeostasis (38). TNF-α has been shown to promote the activation of MCP-1 (39); however, we did not observe a significant difference between the studied groups. *In vitro* studies with macrophage cells showed that increased MCP-1 levels may also be triggered in the presence of palmitic acid and LPS. Palmitic acid is one of the most common saturated fats found in food or synthesized through *de novo* lipogenesis, while high LPS has been reported in diabetic and obese subjects (40, 41). Previous studies have evaluated the association between caspase-1 and the gut microbiota due to its closeness with caspase-4 in humans, and their possible canonical and non-canonical activation mediated by infections, mainly by LPS-producing bacteria (42, 43).

### A higher presence of microbial PAMP producers was detected in MetS and T2DM study groups

We report a significant rise of specific cytokines among T2DM pediatric subjects related to increased proinflammatory activity and gut permeability, with pathogenic gut microbial communities being a possible causal factor. LPS, a main outer membrane bacterial component, is just one of the many PAMPs that may trigger the innate immune system. Studies have shown that LPS can potentially promote the activation of IL-6 and IL-8 through DNA methylation (40).

In the present study, analysis of the gut microbial taxonomic composition of the three groups showed a significant increase in the presence of LPS-producing enterobacterial species (44) among individuals with MetS and T2DM (Figure 2). *Enterobacter, Klebsiella, Citrobacter, Proteus,* and *Kosakonia* spp. are some of the most relevant enteric pathogenic bacteria that naturally inhabit the human gut (45). *Klebsiella pneumoniae, Enterobacter roggenkampii,* and *Enterobacter hormaechei* were statistically higher in the MetS and T2DM study groups compared with healthy controls (Figure 4). In the case of MetS individuals compared to the healthy group, enterobacterial communities belonging to *Citrobacter* (i.e., *Citrobacter freundii* and *Citrobacter* sp. SL156) were significantly higher (Supplementary Figure S3). Species belonging to the Enterobacteriaceae family, which in turn belong to the Proteobacteria phylum, have been positively related to obesity and MetS in Mexican children (46, 47), and have been also found in obese children from Spanish (48) and American (49) cohorts.

In comparison with healthy subjects, MetS and T2DM groups had higher flagellated microbial communities, where the main component flagellin is also considered a PAMP (Figure 4). Flagellin can activate TLR5, a family of Toll-like receptors on the basolateral side of intestinal epithelial cells, indicating when this bacterial component has crossed the gut epithelia. Additionally, this family of receptors has also been expressed in immune cells such as monocytes and immature dendritic cells (50). In this study, *Proteus mirabilis* was significantly higher in T2DM individuals compared to the MetS group*. Pseudomonas aeruginosa* was more abundant in the MetS group compared to healthy and T2DM individuals, and *Citrobacter freundii* in the MetS study group was higher compared to healthy controls. A common characteristic of these microorganisms is their capacity to produce flagellin (51–53).

It is important to consider that detecting an increased presence of bacterial PAMP producers in MetS and T2DM individuals is insufficient to prove the role of these communities in the host's immune system, and its potential role in the development of metabolic disorders (54). Nevertheless, in the present study, a significant overrepresentation of genes involved in flagellin formation, NOD-like receptor signaling pathway, bacterial secretion system, biofilm formation, and production of virulence factors in MetS and T2DM subjects may imply that pathogen colonization and dysbiosis were taking place in the gut of these subjects. These findings are consistent with previous studies (55). However, and surprisingly, none of the mentioned bacterial species related to LPS and flagellin production have shown a significant correlation with the cytokine profile of the studied individuals. Further analyzes with a larger sample size is required.

Correlational studies between clinical metadata and taxonomic composition evidenced the possible relevance of viruses in the activation of the innate immune system. It is worth noting that not only bacterial components serve as PAMPs. It has been reported that virus-derived nucleic acids may also activate receptors that recognize these patterns (56). *Jiaodavirus* (Myoviridae family) was significantly higher in T2DM subjects and was significantly and positively correlated to MCP-1, IL-10, and IL-18 cytokines (Table 4 and Supplementary Figure S8). *Jiaodavirus* is a DNA bacteriophage that infects *Klebsiella* spp. (57) and its presence can be justified by the significantly increased abundance of this bacterial species in MetS and T2DM study groups. The Inoviridae family is another viral group that showed positive associations with proinflammatory activity since it strongly correlates with CRP levels, MCP-1, IL-10, and IL-18. Both *Jiaodavirus* and the Inoviridae family are DNA bacteriophages already reported to be found in the gut microbiome (58).

The Retroviridae and Rudiviridae families also showed inverse correlations with the host's cytokine profile. Notably, Rudiviridae is a bacteriophage family that can infect archaeal species (53), and Retroviridae has been detected in the gut of subjects with inflammatory bowel disease (IBD). Previous studies offer evidence of how human enteric viruses may benefit from gut bacterial communities using their surface polysaccharides to promote their pathogenesis and infectivity rate (59). Additional studies are needed to further elucidate the role of the virome in human health and in the development of metabolic diseases.

### Increased taxonomic and functional traits indicate a possible loss of the gut's hypoxic environment in subjects with MetS and T2DM

Previous studies have shown that a healthy gut is characterized by a hypoxic environment that promotes colonization by anaerobic microorganisms, which in turns helps maintain its homeostasis (60). Several obligate anaerobic bacteria have been involved in the production of short-chain fatty acids such as butyrate, which serves as an energy source for colonocytes (61). In our study, the abundance of obligate anaerobic bacteria was significantly higher in healthy individuals compared to the MetS and T2DM groups. Some relevant microorganisms were *Bifidobacterium catenulatum, Eubacterium callanderi,* and the *Erysipelatoclostridium, Shaalia,* and *Actinomyces* genera (Figure 4).

*Bifidobacteria* spp. has already been reported to be statistically abundant in normal-weight Chinese children (62) and healthy Spanish pediatric subjects (48). Specifically referring to *Bifidobacterium catenulatum,* studies in Sprague-Dawley rats have shown that this species coupled with *Bifidobacterium pseudocatenulatum* may confer liver protection, decrease MCP-1, ameliorate gut dysbiosis, and prevent bacterial intestinal translocation (63).

There is a connection between gut microbiota and the liver since these microbial communities are involved in bile acid metabolism. After primary bile acids are released by the liver and reach the small intestine upon the ingestion of food, their deconjugation is conducted by bacterial bile salt hydrolases (BSH) from a wide variety of Gram-positive bacteria and are finally converted into secondary bile acids by other bacterial populations, particularly *Bacteroides, Clostridioides, Lactobacillus, Eubacterium,* and *Escherichia* for their reabsorption and return into the liver (64, 65). When gut dysbiosis occurs, bile acids become dysregulated, making a pool with high proinflammatory and cytotoxic activity that may affect liver function (62). In this study, *Eubacterium callanderi* was significantly higher in healthy pediatric subjects, suggesting that it may contribute to the bile acid metabolism homeostasis.

Previous studies have shown that microorganisms belonging to the *Erysipelatoclostridium* genus are butyrate producers (66). In the present work, this genus had a significant negative relation with CRP levels, triglyceride levels, WHtR, WC percentile, and BMI percentile (Supplementary Figure S8). *Clostridioides innocuum* is one of the species that belong to this genus, which has shown to be significantly higher in healthy conditions. On the other hand *Clostridioides ramosum* has been related to obesogenic effects and symptoms that characterize human MetS (67). Therefore, a more detailed analysis at species level is necessary in order to determine their possible role in the development of MetS and T2DM.

The genera *Schaalia*, *Actinomyces,* and *Bifidobacterium* belong to the Actinobacteria phylum. *S. odontolytica, S. meyeri, A. oris, A. naeslundii,* and *A. viscosus* were the species that resulted significantly higher in healthy controls. Most of these have been previously reported to be abundant in the oral cavity of both children and adults and are considered to be nitrite-producers. It is important to note that nitrite has antimicrobial properties and is correlated with improved systemic blood circulation (68). In this study, *Actinomyces* genera was negatively correlated with all metadata related to abdominal circumference and positively correlated with HDL levels. There were no signs of correlation with systolic blood pressure (SBP) or diastolic blood pressure (DBP) metadata. Furthermore, the Actinobacteria phylum has already been reported to be negatively correlated with BMI in American children (49) and statistically abundant in healthy Spanish children (48).

Gut dysbiosis is characterized by an increase in facultative anaerobe microorganisms, causing colonocytes to shift their energetic process, increasing oxygen levels within the gut and diminishing the abundance of obligate anaerobes, promoting the colonization of facultative anaerobic microorganisms. In the present study, anaerobic pathogenic bacteria were significantly increased within MetS and T2DM individuals. Functional studies offered further evidence of this phenomenon, since genes related to aerobic respiration (i.e., oxidative phosphorylation, glycolysis/gluconeogenesis, and pyruvate metabolism) were significantly higher in both study groups. Additionally, the copper resistance gene was overrepresented in MetS subjects. Copper has shown to be crucial for β-proteobacteria (enteric bacteria belong to this group) to conduct aerobic respiration since cytochrome oxidases aa3 and cbb3 need it for this process (69, 70). In this case, the presence of cbb3-type cytochrome oxidase was overrepresented, which supports the findings presented here.

After analyzing the overrepresented and underrepresented genes belonging to these metabolic pathways in each of the study groups, the present work explored the hypothesis that the gut microbiome from MetS could be functionally inclined to present an increased production of acetyl-CoA, oxaloacetate, and pyruvate. Pyruvate dehydrogenase was underrepresented. Meanwhile, genes such as coenzyme A synthase and oxaloacetate decarboxylase were overrepresented. According to reported literature, these increased levels of acetyl-CoA, oxaloacetate, and pyruvate may favor the production of branched-chain amino acids (BCAAs) in Gram-negative bacteria; and pantothenate (vitamin B5) and branched-chain fatty acids (BCFAs) in Gram-positive bacteria (71). BCAAs cannot be synthesized by humans; they are usually obtained from exogenous sources (i.e., diet) or by their synthesis mediated by gut microbiota (72). An increased presence of BCAAs in systemic circulation has been found in individuals with different cardiometabolic conditions correlated with factors that could result in MetS and T2DM, such as IR, lipogenesis in adipose tissue, and mitochondrial overload in the skeletal muscle (73); all of which may result in the host's impaired glucose and lipid metabolism. In this work, the high-affinity BCAA transport protein was overrepresented in MetS subjects; meanwhile, in T2DM individuals, the BCAA transporter mediated by the ATP gene was surprisingly underrepresented (Supplementary Tables S3, S4). BCAA transporters are relevant since they have been related to the promotion of pathogenic virulence during infection. Some examples of species that require BCAA biosynthesis for their infection are *Klebsiella pneumoniae* and *Pseudomonas aeruginosa* (71), where increased virulence factors were also found to be overrepresented in the functional potential module, and these species also resulted in being significantly higher in MetS and T2DM subjects when compared to the healthy group (Figure 4). Additionally, it was observed that *Pseudomonas aeruginosa* was significantly and positively related to BMI percentile and hip circumference; meanwhile, *Klebsiella pneumoniae* was positively correlated with BMI percentile, hip circumference, and insulin levels (Supplementary Figure S8). Interestingly, *Klebsiella oxytoca* showed even stronger positive correlations with this metadata and with other ones such as weight, triglycerides, fasting glucose, insulin, HOMA, and CRP levels compared to the *K. pneumoniae*, making it promising to study further. In previous studies, *Klebsiella* spp*.* has already been found to be more abundant in obese Chinese children with nonalcoholic liver disease (74) and *Pseudomonas aeruginosa* in Japanese adults with T2DM that were not taking metformin (75).

Other significant species belonging to the Enterobacteriaceae family, in addition to *Klebsiella* spp., that were found to be relevant in gut dysbiosis also showed to be positively correlated with relevant clinical metadata related to the progression of MetS, including *Proteus mirabilis* (LDL levels); *Enterobacter roggenkampii* (BMI percentile); and *Enterobacter hormaechi* (BMI percentile, WC percentile, hip circumference, WHtR, triglycerides, and cholesterol levels). Surprisingly, there were no relevant correlations related to IR.

### Taxonomic and functional potential studies reveal a possible increase in aerobic respiration and denitrification pathways in individuals with MetS and T2DM

Prior works have reported that cbb3-type cytochrome c oxidase, which was overrepresented in the MetS group (Supplementary Table S3), does not participate exclusively in aerobic respiration but also in the bacterial denitrification pathway mainly conducted by facultative anaerobes with the help of ubiquinol oxidase (76). In this study, the ubiquinol oxidase gene was overrepresented in the samples from MetS individuals, which suggests that this pathway is occurring. Results from differential abundance analysis of KEGG pathways, obtained from healthy, MetS, and T2DM study groups, indicated that the nitrogen metabolism pathway was the only one significantly increased in MetS and T2DM individuals when compared with the healthy group. Additionally, this pathway was also significantly overrepresented in the T2DM study group compared with MetS individuals, inferring that this pathway may be progressively increasing from a healthy state until reaching a T2DM condition. It is noteworthy to mention that when gut dysbiosis takes place and colonocytes shift their energetic metabolism into aerobic respiration rather than β-oxidation, levels of nitrate $\left(NO_{3}^{-}\right)$ also start to increase in the intestinal lumen (60), which is the fundamental substrate of the denitrification pathway and other pathways conducted within the gut.

The aforementioned observations and interpretations can also be strengthen by the increased presence of facultative anaerobe bacteria within MetS and T2DM individuals (Figure 4), which has been already discussed. Previous publications have already addressed the main nitrogen pathways conducted in the human gut, of which the denitrification, dissimilatory nitrate $\left(NO_{3}^{-}\right)$ reduction to ammonia (NH_4_), and the non-enzymatic conversion of nitrite-to-nitrite oxide (NO) pathways are mainly conducted by gut microbiota, whereas the endogenous L-arginine synthase pathway is conducted by the intestinal epithelial cells. In the denitrification pathway, facultative anaerobe bacteria convert $NO_{3}^{-}$ to nitrogen (N_2_), decreasing levels of NO (77). It is relevant to mention that NO levels are essential to the host's homeostasis since this gas migrates into the circulatory system to reach other organs and tissues, causing cardioprotective effects. When NO depletion occurs, there is a loss in cardiovascular homeostasis that also leads to increased superoxide anions $\left(O_{2}^{-}\right)$, which may promote mitochondrial dysfunction, loss of gut permeability, and leukocyte diapedesis (78).

The present study found that several strict obligate species belonging to the Actinobacteria phylum were depleted in individuals with MetS and T2DM, clearly demonstrating alterations in the beneficial NO pool in the gut. According to literature, these microorganisms are nitrite $\left(NO_{2}^{-}\right)$ producers using $NO_{3}^{-}$ as substrate (68), with $NO_{2}^{-}$ being a precursor of NO production through the non-enzymatic pathway. Interestingly, the significantly increased presence of specific lactic acid bacteria (which are also considered to be facultative anaerobe microorganisms) was detected in T2DM groups compared to the healthy condition. They can also be involved in the nitrogen metabolism of the host by conducting NO production, acidifying the gut environment (79). *Weissella cibaria* and *Lactobacillus ruminis* were the species that resulted significantly higher in T2DM individuals. Notably, no lactic acid bacteria were highly present in the MetS group compared to the healthy condition.

According to correlational studies, *Weissella cibaria* and *Lactobacillus ruminis* showed an interesting positive association with multiple clinical metadata that corresponded to MetS risk factors such as weight, BMI, WC percentile, WHtR, hip circumference, TG levels, FBG, insulin levels, HOMA-IR, and CRP levels (Supplementary Figure S8). Additionally*, Weissella cibaria* was negatively correlated with HDL levels. Associations that may suggest that these microorganisms can impact the MetS prevalence. However, previous studies have reported that *Weissella cibaria* confers beneficial properties to the host (i.e., cholesterol reduction and prevention of LPS-induced proinflammatory activity) (80). Findings of *Lactobacillus ruminis* in the unhealthy subjects were consistent with previously reported studies, since it was also significantly higher in adult Indians diagnosed with T2DM (81) and significantly lower in healthy young Indonesians compared to adults (82). Additionally, studies conducted on American children showed that *Lactobacillus* spp. was more abundant in obese individuals (49). Therefore, the present work suggests that impairments in nitrogen metabolism may result in undesirable gut microbial composition changes influencing the onset of relevant MetS risk factors that can end in the progression of T2DM.

### Dominant gut microbial phyla are not determinative biomarkers to infer MetS and T2DM risk in Mexican pediatric subjects

Nowadays, the Firmicutes/Bacteroidetes (F/B) ratio has gained popularity since it has been proposed as a potential biomarker to determine gut dysbiosis and predict the risk of developing obesity. However, it is still controversial due to conflicting evidence (83). In the present study, the F/B ratio was calculated for each of the study groups, but with no significant difference among them (Supplementary Table S2); however, data showed a decreasing trend from healthy to a MetS state and then an increasing trend for patients diagnosed with T2DM. Results, therefore, contrasted with other pediatric studies where healthy and obese conditions were analyzed. Studies in Italian children obtained a significantly higher F/B ratio in obese subjects compared to normal-weight children (84). Conversely, in the case of Swiss and Kazakh children, both studies registered a lower F/B ratio in obese children compared to normal-weight individuals (85, 86). Moreover, in agreement with the present study results, no differences were perceived in Mexican children in a study conducted by López-Contreras et al. (2017), which reported no significant difference in the F/B ratios of obese and normal-weight children (87). Other pediatric microbiota studies by Mendez-Salazar et al. (2018) focused in undernourished, normal-weight, and obese Mexican children, for which the F/B ratio showed to be greater in the first study group. The authors attributed the results to diet components in undernourished subjects that were rich in sugars and low in fiber (46). Similarly, it has been established that diets rich in glucose and sucrose increase this ratio (88).

Evident limitations in the F/B ratio as a biomarker of metabolic disease exist, which may be attributed to oversimplification, since it is important to note that there are gut microbial communities belonging to the Bacteroidetes and Firmicutes phyla that may have positive or negative impacts on human health. For instance, in this study, *Alistipes shahii* was shown to be significantly more abundant in MetS and T2DM individuals compared to the healthy group. This species belongs to the Bacteroidetes phylum, and in this work, it was positively correlated with weight, BMI, waist circumference, WHtR, triglyceride levels, FBG, insulin levels, HOMA-IR, and CRP levels (Supplementary Figure S8). Moreover, this species has already been reported in Mexican children but with no significant difference in obese subjects compared to healthy ones (89). In contrast, in Chinese adults, it was statistically abundant in T2DM patients (90), making it promising as a potential biomarker of the disease. On the other hand, lactic acid bacteria and *Erysipelatoclostridium* genus belong to the Firmicutes phyla in which several species from the lactic acid bacteria have shown to have positive correlations with MetS risk factors, while *Erysipelatoclostridium* spp. showed negative correlations. Additionally, the *Acidaminococcus* genus, which also belongs to Firmicutes, was significantly increased in T2DM individuals when compared to healthy and MetS subjects. Furthermore, it was significantly and positively correlated with cholesterol and triglyceride levels, indicating its possible relevance in fatty acid metabolism. Scarce information has been published about this genus. Still, it was abundant in obese Italian adults (91) and malnourished children, correlated with stunting (92). Evidence suggests that changes in the abundance of most dominant gut microbial phyla within MetS and T2DM individuals, specifically Firmicutes and Bacteroidetes, do not predict the risk of developing the characteristic traits of MetS that promote the progression to T2DM.

## Conclusions

According to the findings of this shotgun metagenomic analysis, it can be concluded that relevant gut microbial species of the studied individuals are significantly associated with MetS and T2DM. Herein, fluctuations in the abundance of enteric bacteria, specific viral communities, and strict anaerobes were relevant to gut homeostasis. The gut microbial dysbiosis observed in the studied subjects promoted the loss of an intestinal hypoxic environment, increased bacterial nitrogen metabolism, and contributed to a higher production of PAMPs. All these alterations have been linked to increased levels of BCAAs and gut permeability, loss of cardiovascular homeostasis, increased mitochondrial oxidative stress, and increased proinflammatory activity. These metabolic imbalances may affect the intermediate metabolism of the host, possibly causing the onset of MetS risk factors that could contribute to the progression of T2DM if these conditions are not treated promptly. Despite this, genomic approaches used here are not sufficient to thoroughly study gut microbiota–host interactions. Other “omic” technologies (i.e., metabolomics, metatranscriptomics, and metaproteomics) and conventional techniques are still needed to validate these results.

Limitations of this study must be considered when interpreting the results, as it disregarded the gut microbial effect of administered medications within the MetS and T2DM study groups (metformin and insulin) and the dietary habits of each of the subjects involved. Nonetheless, it is one of the few studies conducted in MetS and T2DM pediatric individuals and one of the few works in which shotgun metagenomics was used to study the whole gut microbial taxonomic composition, without restricting it to bacteria, and goes further into possible changes in its functional potential.

## Data Availability

The datasets presented in this study can be found in online repositories. The names of the repository/repositories and accession number(s) can be found below: https://www.ncbi.nlm.nih.gov/bioproject/PRJNA821541/, PRJNA821541.
