# Supplementary material for: Gut microbial composition and functionality of school-age Mexican population with metabolic syndrome and type-2 diabetes mellitus using shotgun metagenomic sequencing
Source: Front Pediatr. 2023 May 31;11:1193832. doi: 10.3389/fped.2023.1193832 (PMC10277889; doi:10.3389/fped.2023.1193832)
Supplement: Supplementary file 1 [file Datasheet1.pdf]

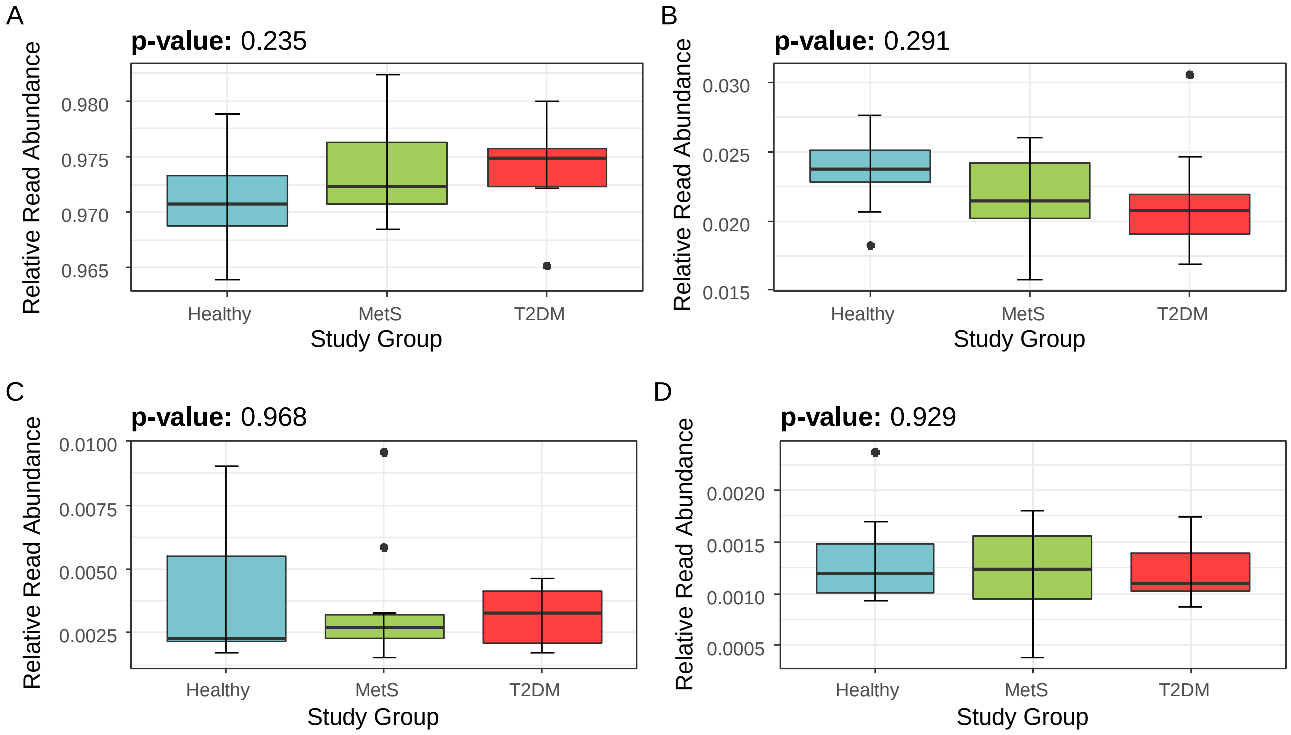

Supplementary Figure 1. Gut microbiota's relative read abundance at domain level: Boxplots show the relative read abundance obtained from healthy, MetS, and T2DM subjects. Median values and interquartile ranges are indicated in the plot. One-way ANOVA test (parametric) or Kruskall-Wallis (nonparametric) tests were used, and significance was established as p-value ≤ 0.05: ***a)*** Bacteria, ***b)*** Eukarya, ***c)*** Archaea, ***d)*** Virus.

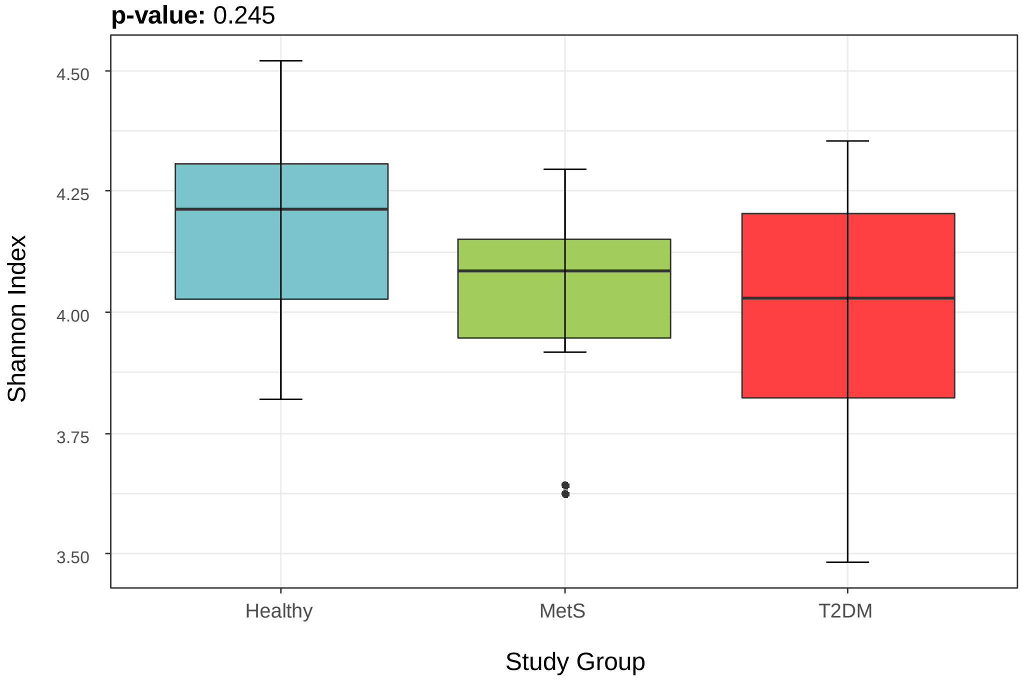

Supplementary Figure 2. Gut microbiota's alpha-diversity at the species level in Mexican pediatric patients: Boxplots show the Shannon index obtained from healthy, MetS, and T2DM subjects. Median values and interquartile ranges are indicated in the plot. One-way ANOVA test was used, and significance was established as p-value $\leq$ 0.05.

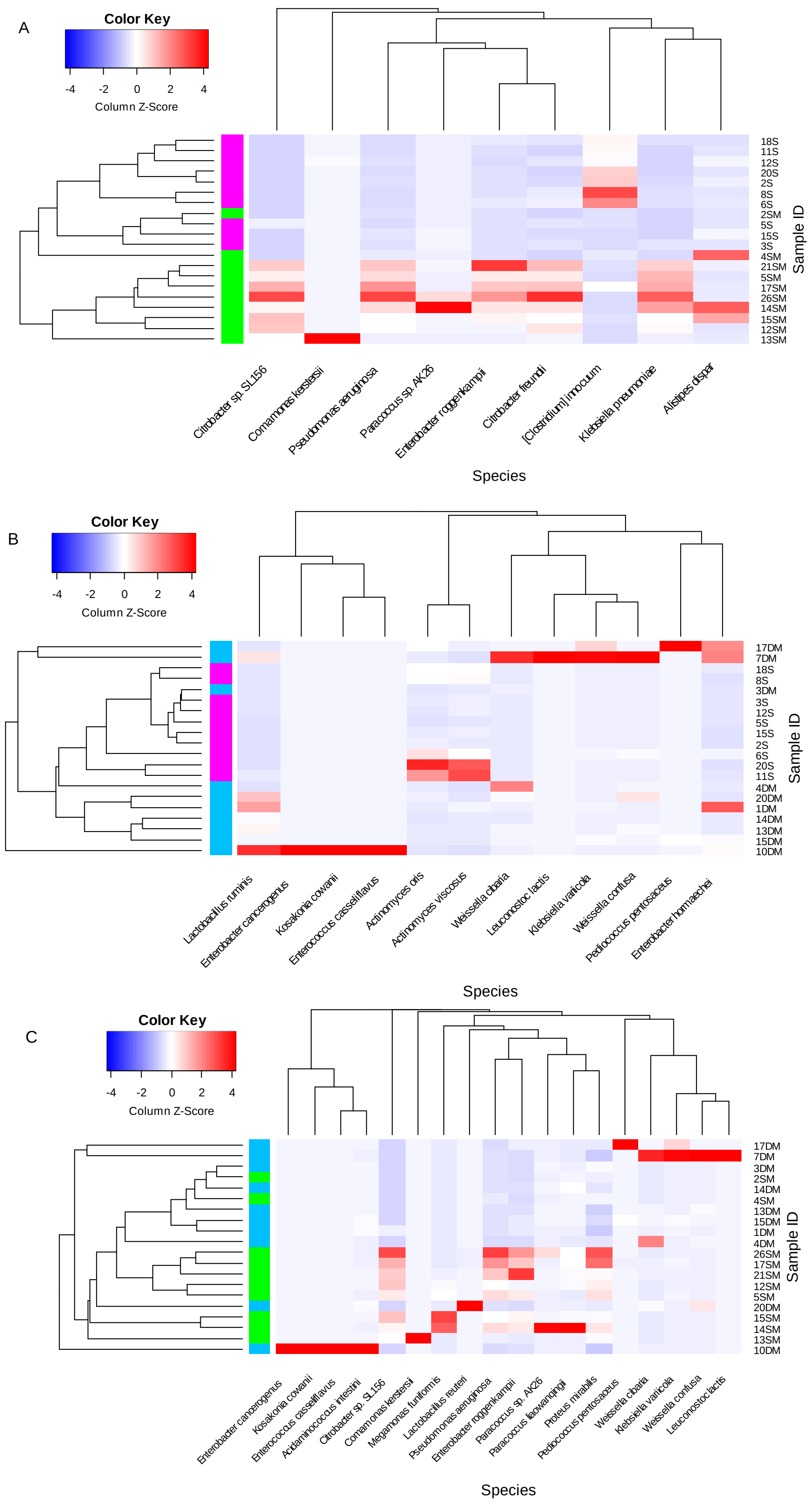

Supplementary Figure 3. Statistically significant microorganisms at species level between study groups: a) MetS vs. healthy; **b)** T2DM vs. healthy; **c)** T2DM vs. MetS. Magenta refers to healthy subjects, green for MetS, and blue for T2DM individuals.

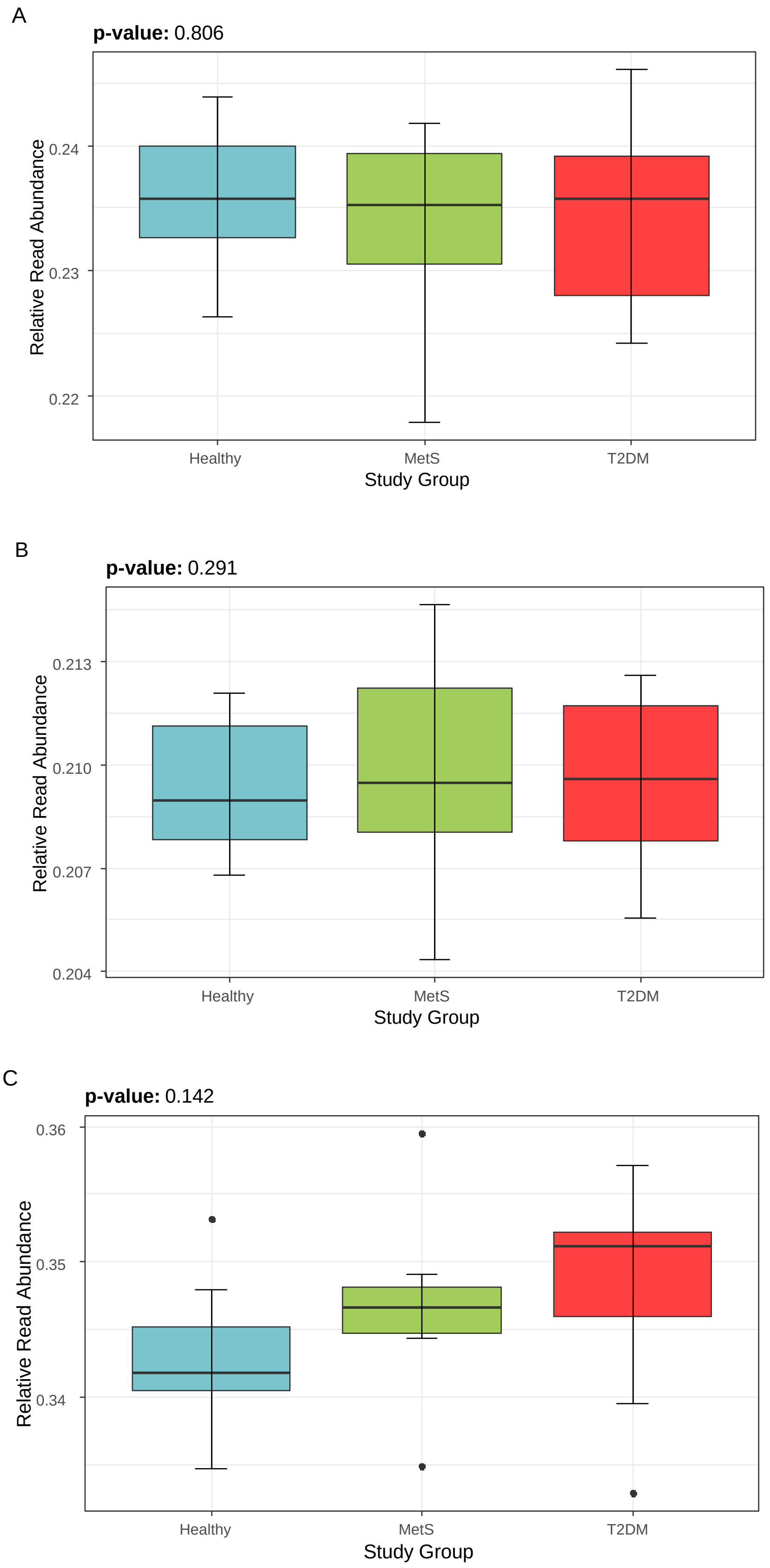

Supplementary Figure 4. Relative read abundance of general gut microbial functional potential categories: Boxplots show the relative read abundance obtained from healthy, MetS, and T2DM subjects. Median values and interquartile ranges are indicated in the plot. One-way ANOVA tests were used, and significance was established as p-value $\leq$ 0.05: ***a)*** Information Storage and Processing, ***b)*** Cellular Processes and Signaling, ***c)*** Metabolism.

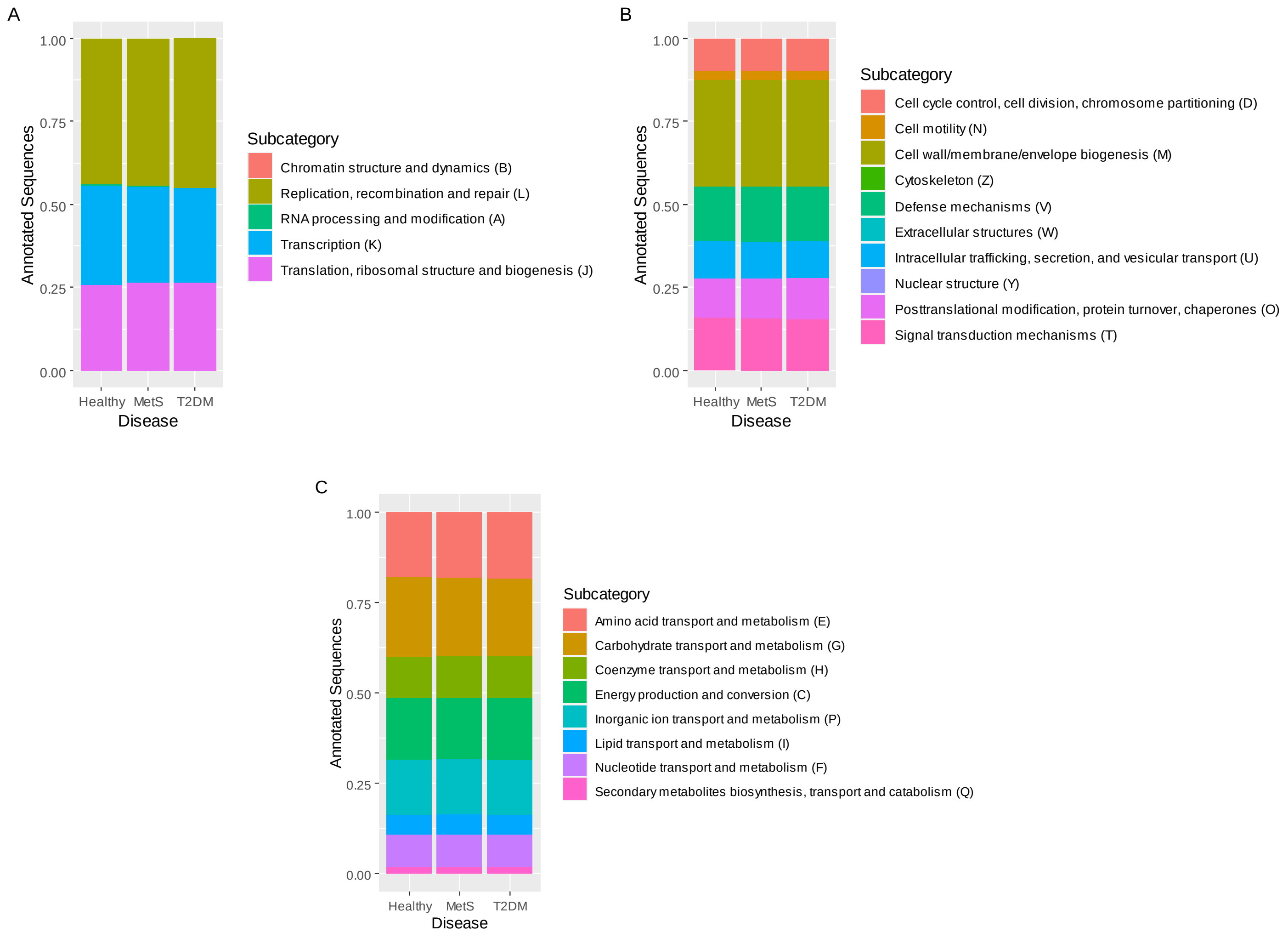

Supplementary Figure 5. Sub-categorical relative abundance from predicted functional potential among each study group: *a)* Information Storage and Processing, ***b)*** Cellular Processes and Signaling, ***c)*** Metabolism.

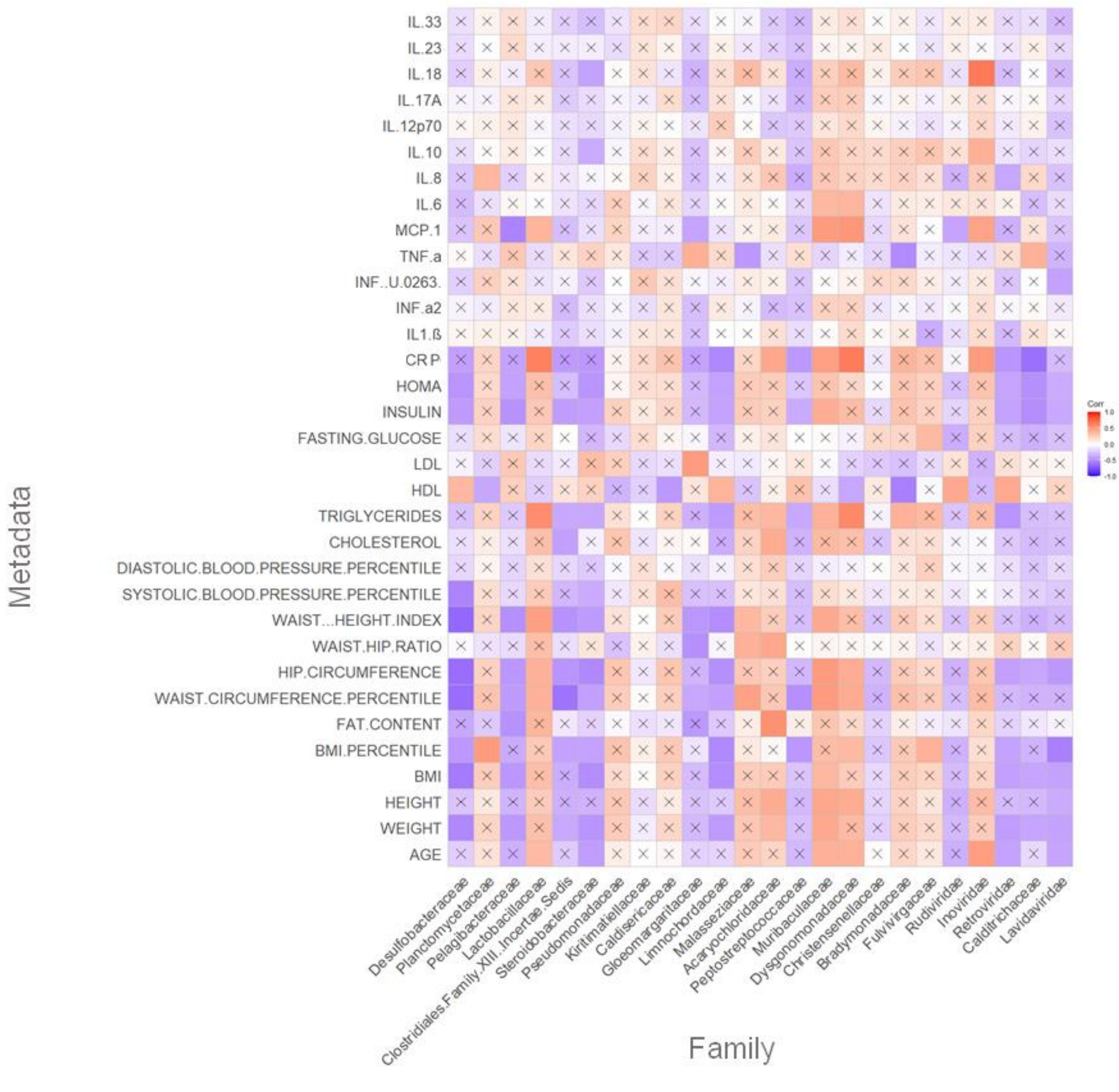

Supplementary Figure 6. Pearson's correlation between metadata and significant families

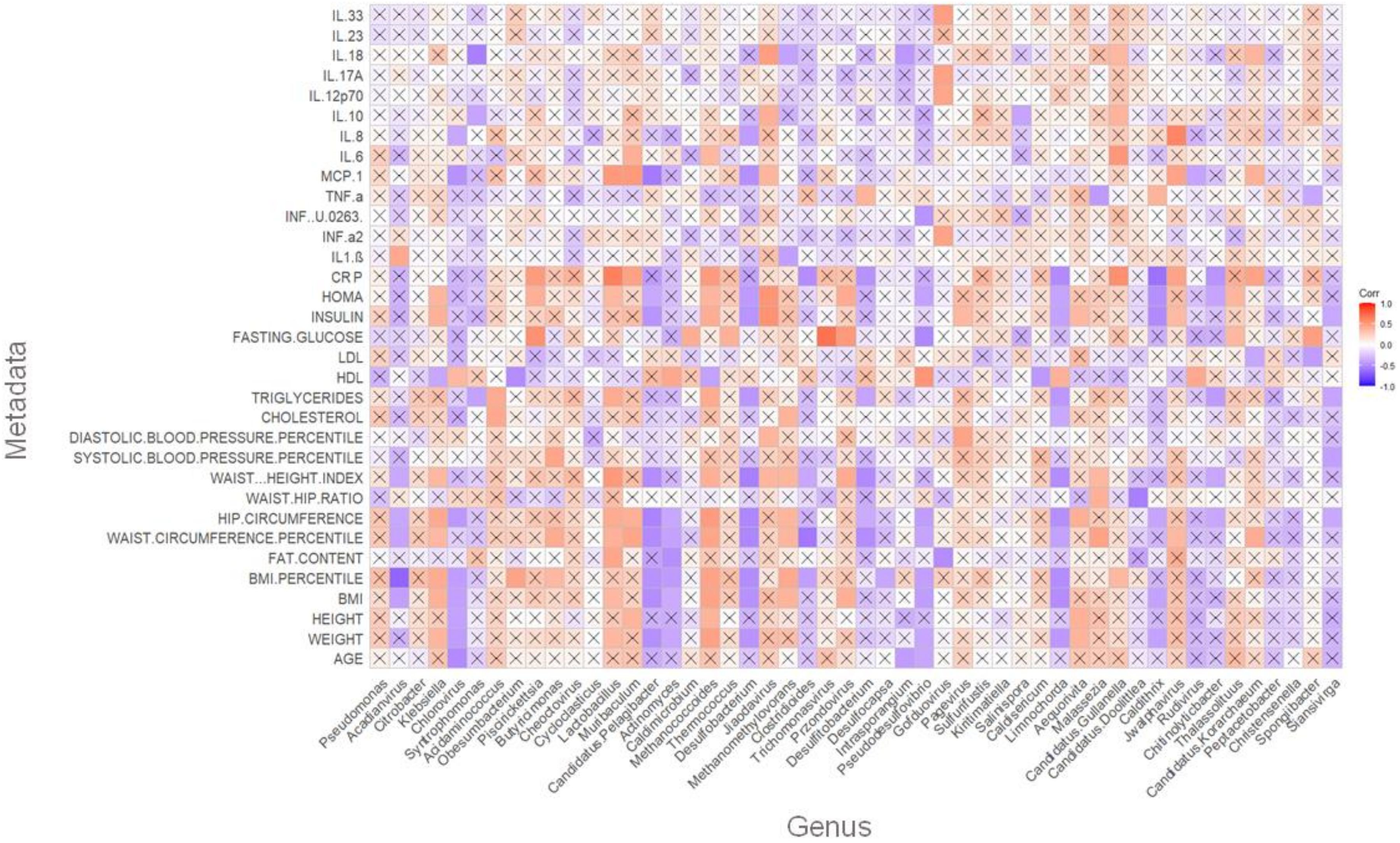

Supplementary Figure 7. Pearson’s correlation between metadata and significant genera

Supplementary Figure 7. Pearson's correlation between metadata and significant genera [Continued]

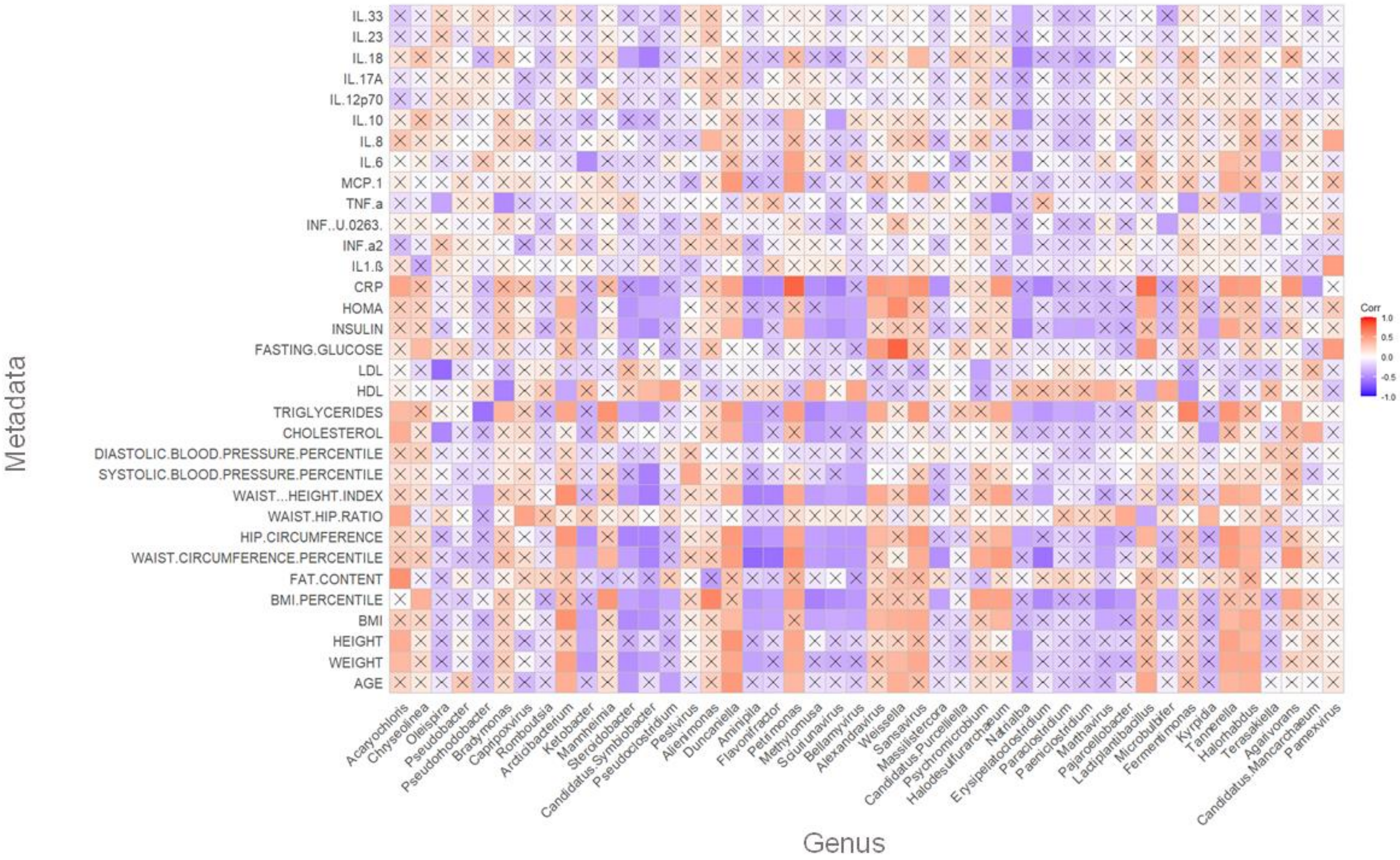

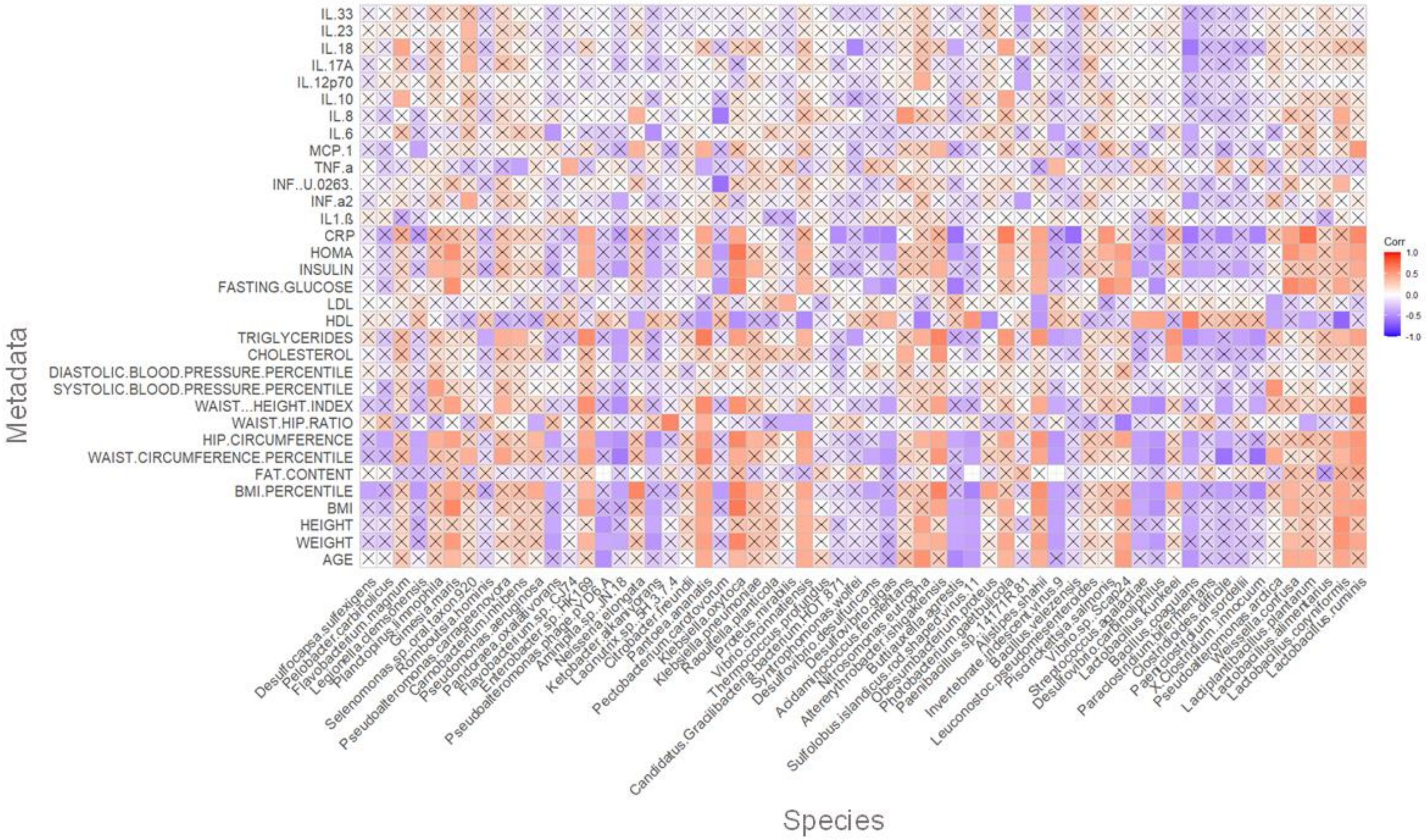

Supplementary Figure 8. Pearson's correlation between metadata and significant species

Supplementary Figure 8. Pearson's correlation between metadata and significant species [Continued]

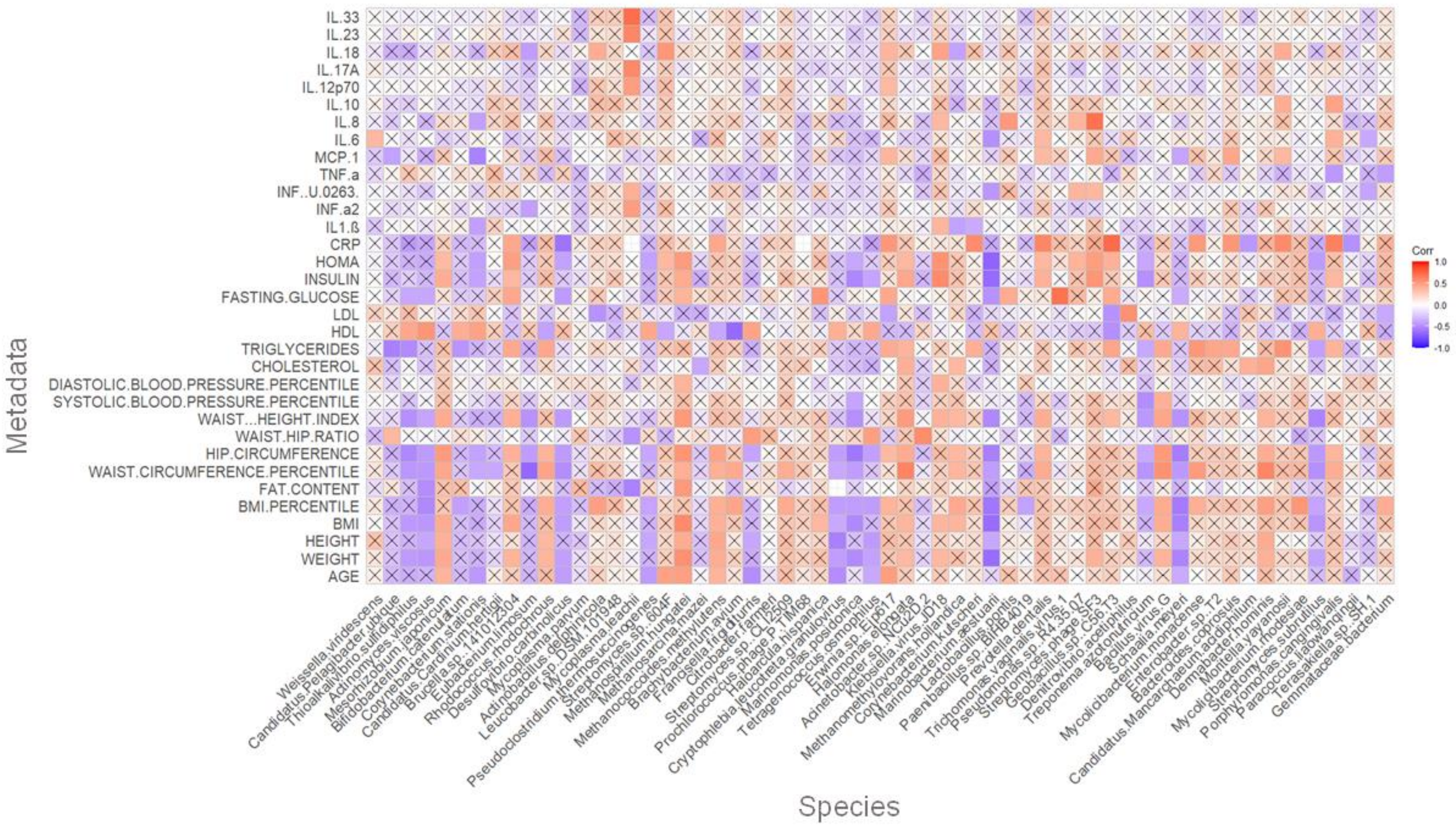

Supplementary Figure 8. Pearson's correlation between metadata and significant species [Continued]

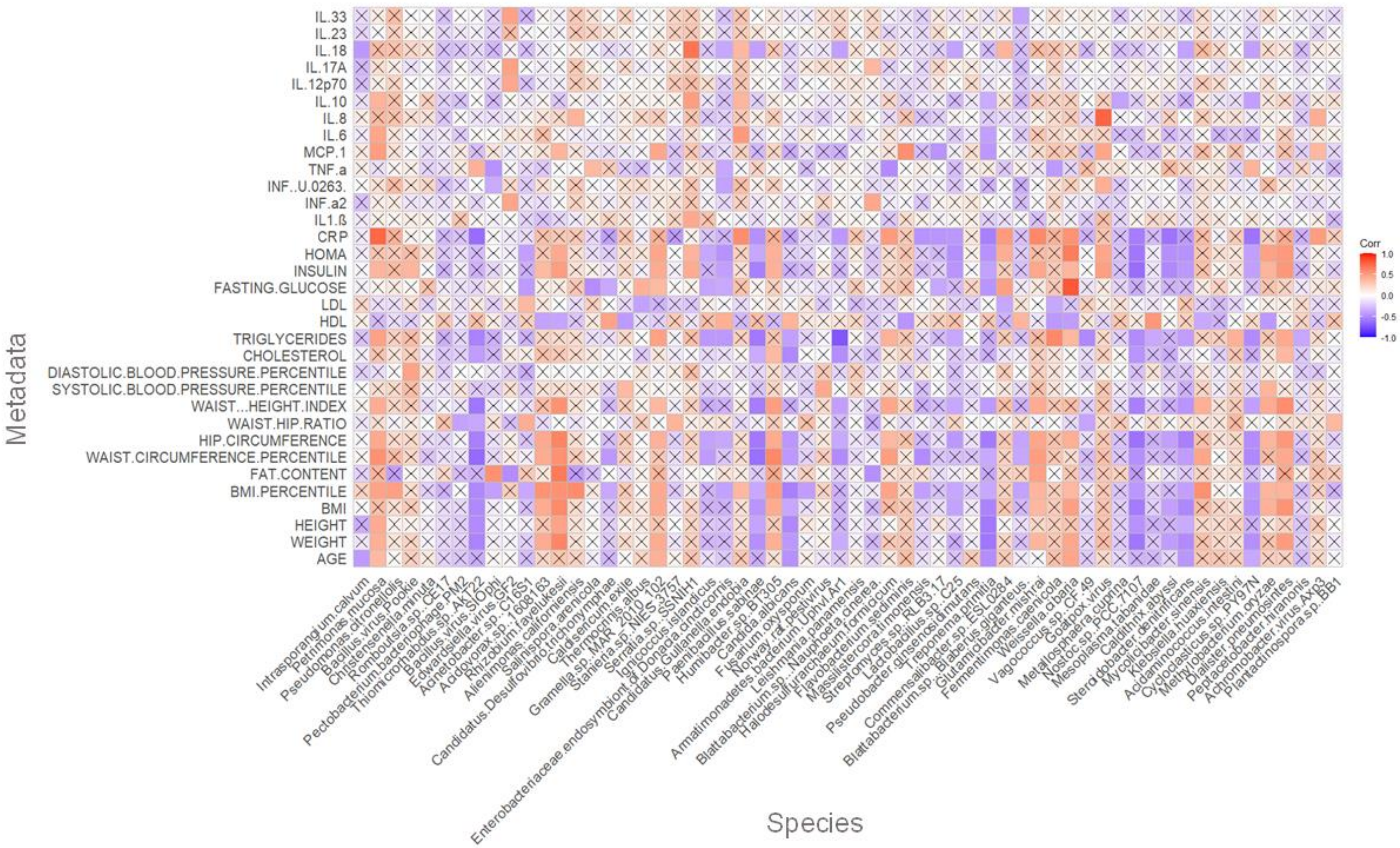

Supplementary Figure 8. Pearson's correlation between metadata and significant species [Continued]

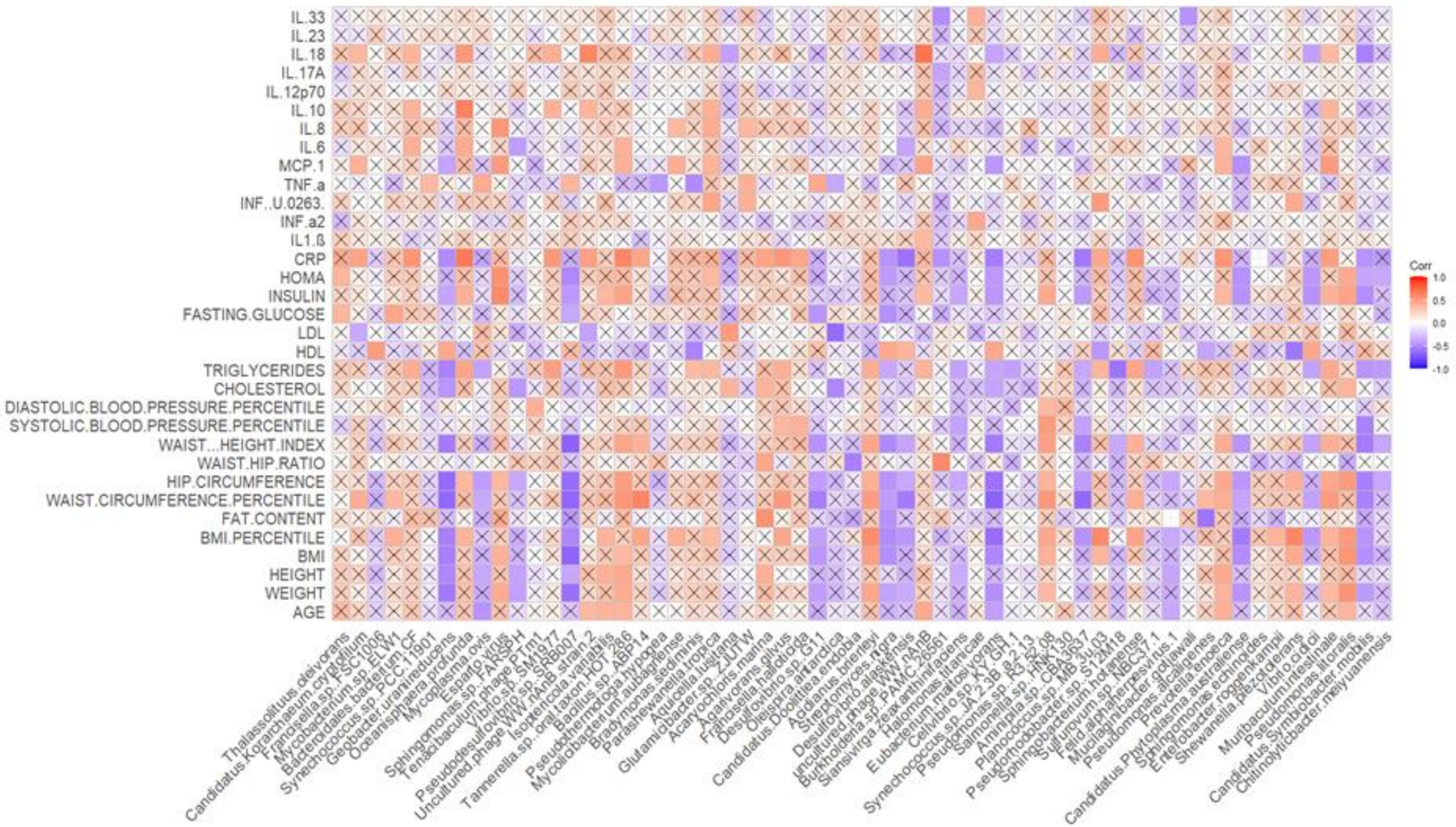

Supplementary Figure 8. Pearson's correlation between metadata and significant species [Continued]

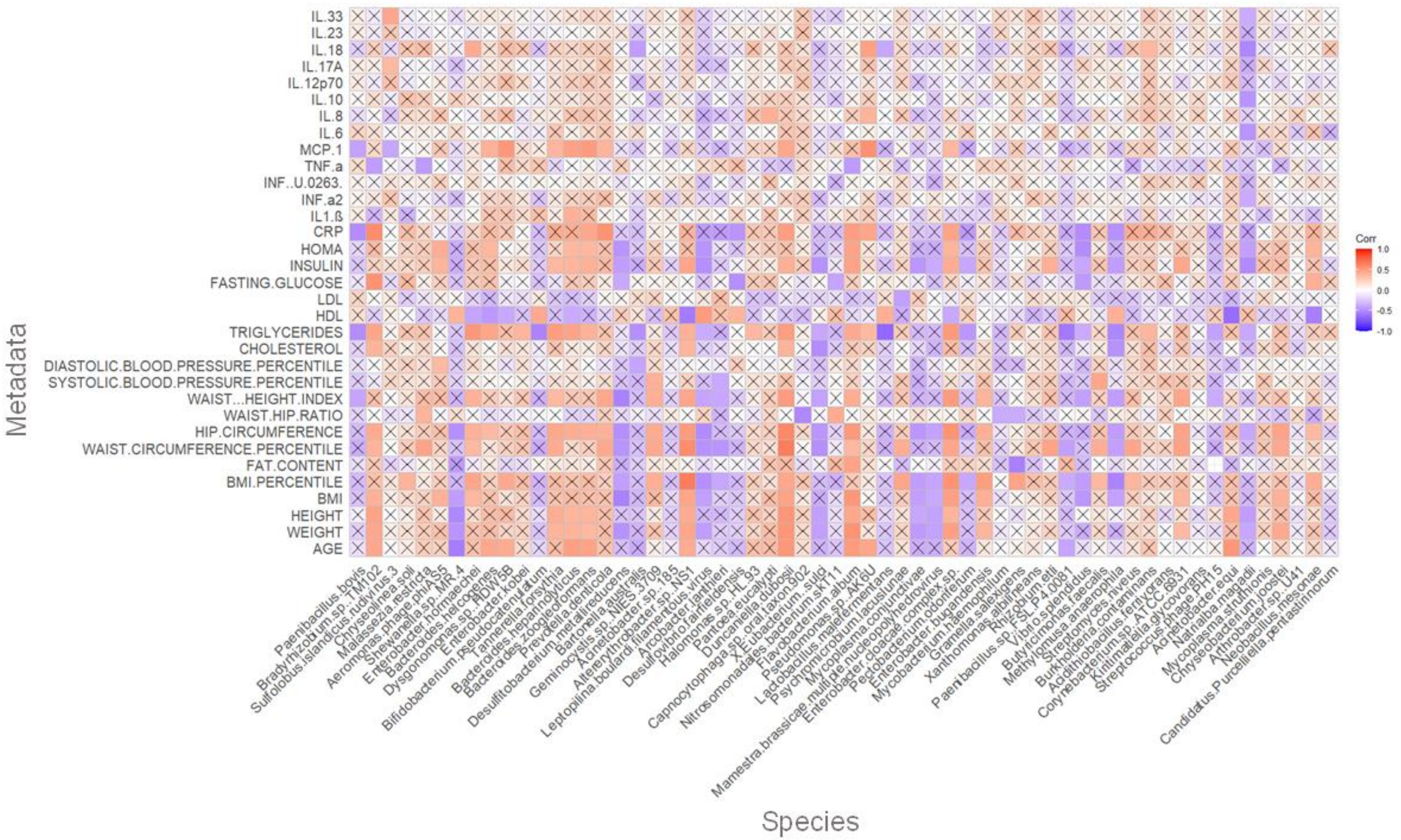

Supplementary Figure 8. Pearson's correlation between metadata and significant species [Continued]

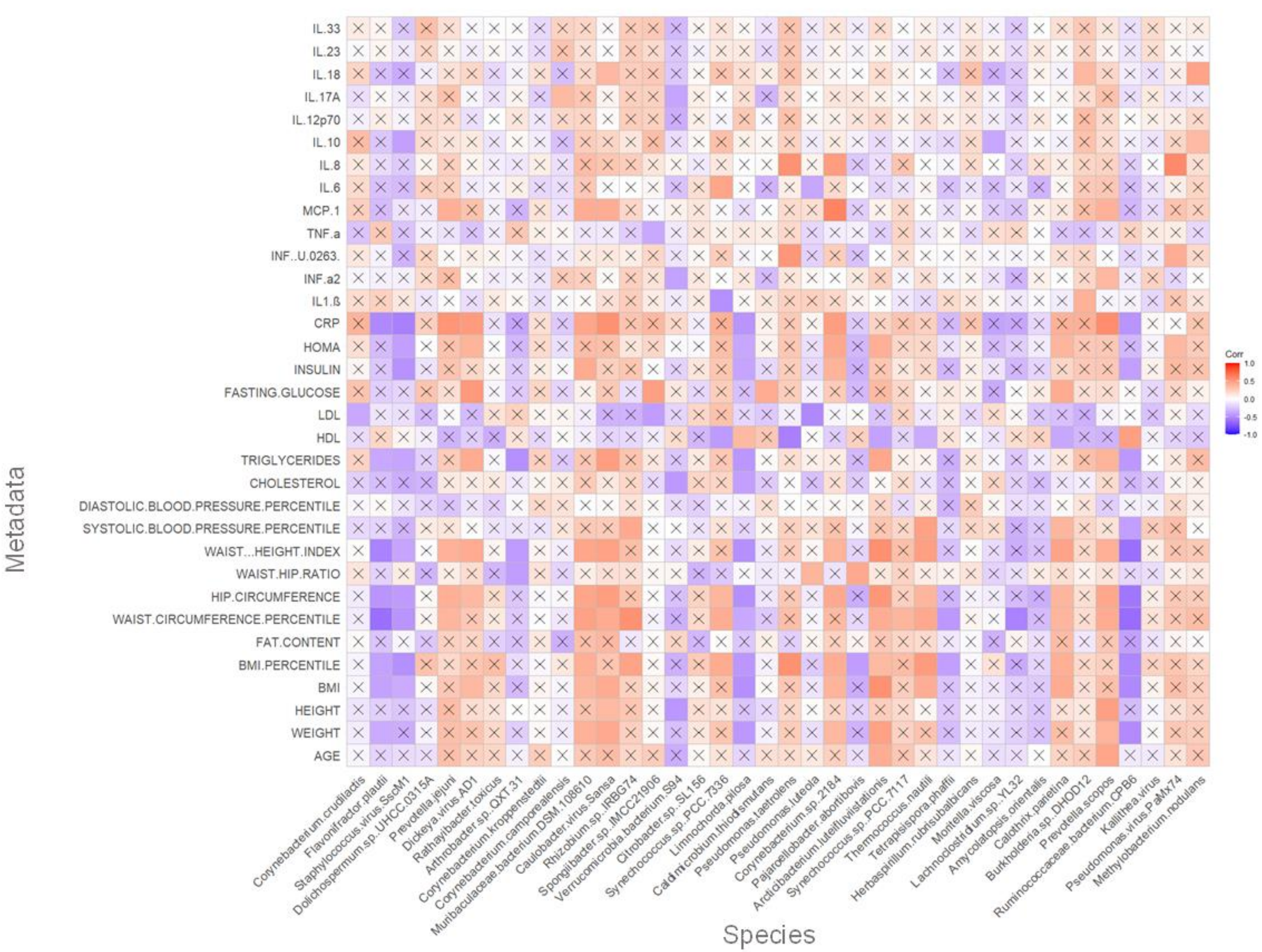

Supplementary Table 1. Raw read sequencing and annotation results

Health Condition	n	Total Initial Reads	Total Clean Reads	Human Reads (%)	Total # Contigs	Taxonomic Classification (%)	Total Predicted Genes	EggNOG Annotation (%)
Healthy	10	407,284,160	407,046,164	0.06	4,711,372	60.60	6,904,218	18.01
MetS	10	394,222,438	393,824,048	0.10	5,053,592	61.49	7,005,079	18.12
T2DM	10	408,608,256	408,311,616	0.07	6,152,911	61.07	8,303,423	18.22

Supplementary Table 2. Firmicutes / Bacteroidetes ratio from healthy, MetS, and T2DM Mexican pediatric subjects

Parameter	Healthy (n = 10)	MetS (n = 10)	T2DM (n = 10)	p-value
F/B Ratio	3.881 (3.203 – 4.815)	3.108 (2.874 – 4.181)	3.607 (2.927 – 4.117)	0.475

Data are shown as median (Q25% - Q75%) since it has been shown to be nonparametric data. Kruskall-Wallis test was used, and significance was established as a p-value ≤ 0.05*: ***a*** represents the statistical difference between healthy and MetS, ***b*** between healthy and T2DM, and ***c*** between MetS and T2DM.

Supplementary Table 3. Overrepresented COG/KOG/NOG Orthology genes in MetS and T2DM vs healthy

Study Group	COG/KOG/NOG Description	Possible Chemical and Biological Participation	logFC	SE	p-value	FDR
MetS	Mesenchymal-epithelial cell signaling involved in prostate gland development	Wnt pathway	21.135	3566.331	<0.001	0.004
	Nuclease of the RecB family	DNA recombination and repair	20.882	3146.015	<0.001	0.004
	Oxaloacetate decarboxylase gamma chain	Pyruvate metabolism<br>Benzoate degradation	20.502	2726.228	<0.001	0.004
	FK506 binding protein	Immunosuppresion mechanisms	20.729	3377.021	<0.001	0.014
	Monooxygenase	Oxidorreductase activity (NADH → NAD+)	20.196	2649.128	<0.001	0.016
	Poly-beta-1,6 N-acetyl-D-glucosamine export porin PgaA	Biofilm formation	19.933	2436.801	<0.001	0.022
	Protocatechuate 3,4-dioxygenase, beta subunit'	Aerobic benzoate degradation	20.502	3374.801	<0.001	0.028
	TIGRFAM radical SAM additional 4Fe4S-binding domain	Porphyrin and chlorophyll metabolism	20.335	3313.698	<0.001	0.028
	Deiminase	Arginine biosynthesis	20.017	2693.072	<0.001	0.028
	Catalytic LigB subunit of aromatic ring-opening dioxygenase	Aerobic benzoate degradation	19.883	2614.541	<0.001	0.028
	3,4-dihydroxyphenylacetate 2,3-dioxygenase	Tyrosine metabolism	19.883	2614.541	<0.001	0.028
	Alkanesulfonate monooxygenase	Sulfur metabolism	19.882	2558.230	<0.001	0.028
	Poly-beta-1,6-N-acetyl-D-glucosamine transmembrane transporter activity	Biofilm formation	19.882	2658.583	<0.001	0.029
	PFAM CopG domain protein DNA-binding domain protein	Folding, sorting, and degradation	20.335	3550.059	<0.001	0.031
	Proteasome-type protease	Folding, sorting, and degradation	20.335	3425.539	<0.001	0.031
	Plectin repeat	Intestinal epithelial protection	20.135	3155.627	<0.001	0.031
	Copper resistance protein	Protection against copper toxicity	19.883	2758.491	<0.001	0.031
	Involved in type III protein export during flagellum assembly	Flagellum formation	19.883	2751.241	<0.001	0.031
	ubiquinol oxidase subunit	Oxidative phosphorylation	19.577	2406.296	<0.001	0.031
	Proteins of 100 residues with WXG	Gram-(+) bacteria secretion system	19.547	2383.413	<0.001	0.031
	Antirestriction protein	Bacterial pathogenesis	2.546	0.687	<0.001	0.032
	Cytochrome c oxidase, cbb3-type, subunit II	Oxidative phosphorylation	19.883	2883.875	<0.001	0.035
	PFAM 20S proteasome, A and B subunits	Folding, sorting, and degradation	20.240	3484.115	<0.001	0.036
	Choline dehydrogenase and related flavoproteins	Pentose-Phosphate pathway	19.828	2833.989	<0.001	0.036
	Restriction endonuclease FokI, C terminal	Methylation	2.205	0.572	<0.001	0.038
	Acyl-CoA dehydrogenase N terminal	Fatty acid degradation<br>Branched-chain amino acid degradation	19.933	3067.383	<0.001	0.042
	Catalyzes the sequential removal of 2 amino-terminal arginines from alkaline phosphatase isozyme 1 to form isozymes 2 and 3	NOD-like receptor signaling pathway	20.502	4131.211	<0.001	0.043
	Cellulose synthase operon protein YhjQ	Starch and sucrose metabolism	2.546	0.731	<0.001	0.043
	High-affinity branched-chain amino acid transport protein (ABC superfamily)	ABC transporters	19.324	2315.886	<0.001	0.045
	COG2993 Cbb3-type cytochrome oxidase, cytochrome c subunit	Oxidative phosphorylation	20.135	3489.075	<0.001	0.046
	Part of the ABC transporter complex FbpABC involved in Fe(3) ions import. Responsible for energy coupling to the transport system	ABC transporters	1.002	0.223	<0.001	0.047
	Hemolysin activation secretion protein	Bacterial secretion system	2.871	0.917	<0.001	0.048
	Coenzyme A synthase	Glycolysis and Gluconeogenesis<br>Piruvate metabolism	19.324	2360.504	<0.001	0.048
	Catalyzes the deimination of N-formimino-L-glutamate to ammonia and N-formyl-L-glutamate	Histidine metabolism	19.883	3148.642	<0.001	0.049
	ABC-type dipeptide oligopeptide nickel transport systems, permease	ABC transporters	1.890	0.492	<0.001	0.049
	Flavin Reductase	Riboflavin metabolism	20.135	3596.859	<0.001	0.049

***logFC = log fold-change; SE = standard error; FDR = false discovery rate**

Supplementary Table 3. Overrepresented COG/KOG/NOG Orthology genes in MetS and T2DM vs healthy [Continued]

Study Group	COG/KOG/NOG Description	Possible Chemical and Biological Participation	logFC	SE	p-value	FDR
T2DM	phosphocarrier protein HPR	Phosphotransferase system	20.964	3654.197	<0.001	0.008
	Zn-dependent alcohol dehydrogenases, class III	Aldehyde detoxification	20.964	3713.622	<0.001	0.008
	TIGRFAM Nicotinamide mononucleotide transporter PnuC	B-type vitamin transporter	20.376	3280.038	<0.001	0.026
	LPP20 lipoprotein	Flagellar assembly	19.559	2261.990	<0.001	0.031
	conjugal transfer protein TraG	DNA transfer	20.406	3481.899	<0.001	0.031
	Acetyl-CoA carboxylase biotin carboxylase subunit	Fatty acid biosynthesis<br>Pyruvate metabolism<br>Propanoate metabolism<br>Carbon fixation	20.165	3107.238	<0.001	0.031
	COGs COG0641 Arylsulfatase regulator (Fe-S oxidoreductase)	Poorly characterized	20.376	3542.605	<0.001	0.031
	ATP-dependent helicase/nuclease subunit A	DNA recombination and repair	19.559	2440.653	<0.001	0.040
	methionine biosynthesis	Bacterial methionine biosynthesis	19.376	2291.531	<0.001	0.043
	Transglutaminase	NH3 production	19.964	3088.501	<0.001	0.043
	Bile acid	Secondary bile acid production	19.713	2750.261	<0.001	0.043
	Binds processed UmuD protein to form functional DNA pol V (UmuD'2UmuC)	SOS response to DNA damage	19.711	2799.941	<0.001	0.043
	COG0466 ATP-dependent Lon protease, bacterial type	Protein quality-control system	19.376	2382.157	<0.001	0.043
	Rubredoxin-like zinc ribbon domain (DUF35_N)	Poorly characterized	20.153	3540.972	<0.001	0.043
	EnpEP protein	Renin-angiotensin system	20.153	3540.972	<0.001	0.043
	YcbB domain protein	Peptidoglycan biosynthesis	19.559	2640.225	<0.001	0.044
	PFAM WbqC-like protein family	Poorly characterized	20.153	3679.010	<0.001	0.047
	Catalyzes the transfer of the formyl group from N- formylglutamate to tetrahydrofolate (THF) to yield 5- formyltetrahydrofolate (5-CHO-THF) and glutamate (Glu). The triglutamate form of 5-CHO-THF (5-CHO-THF-Glu3) can also be used as substrate. It can also catalyzes the transfer of the formimino group from N-formiminoglutamate to tetrahydrofolate (THF) to yield 5-formiminotetrahydrofolate (5-NH CH-THF) and glutamate (Glu). It can replace YgfA to catalyzes the irreversible ATP-dependent transformation of 5-CHO-THF to form 5,10-methenyltetrahydrofolate (5,10-CH THF)	Histidine metabolism	20.153	3679.010	<0.001	0.047
	PFAM tRNA rRNA methyltransferase, SpoU	RNA processing	20.153	3679.010	<0.001	0.047
	Sodium neurotransmitter symporter	Sodium-mediated transport	20.153	3679.010	<0.001	0.047
	Catalytic LigB subunit of aromatic ring-opening dioxygenase	Aerobic benzoate degradation	19.713	2980.594	<0.001	0.048
	3,4-dihydroxyphenylacetate 2,3-dioxygenase	Tyrosine metabolism	19.713	2980.594	<0.001	0.048
	Choline dehydrogenase and related flavoproteins	Pentose-Phosphate pathway	20.025	3491.189	<0.001	0.048
	aluminum resistance	Protection against aluminum toxicity	20.406	4236.363	<0.001	0.048
	PFAM AAA-ATPase-like	ATP binding	19.376	2539.819	<0.001	0.048
	POTRA domain protein, ShlB-type	Bacterial secretion system	20.069	3597.271	<0.001	0.048
	Belongs to the glycosyl hydrolase 17 family	Starch and sucrose metabolism	19.153	2280.496	<0.001	0.048

***logFC = log fold-change; SE = standard error; FDR = false discovery rate**

Supplementary Table 4. Underrepresented COG/KOG/NOG Orthology genes in MetS and T2DM vs healthy

Study Group	COG/KOG/NOG Description	Possible Chemical and Biological Participation	logFC	SE	p-value	FDR
MetS	Major royal jelly protein	Yellow gene family	-21.268	3829.596	<0.001	0.004
	Related to alanyl-tRNA synthetase HxxxH domain	Aminoacyl-tRNA biosynthesis	-20.757	3542.625	<0.001	0.017
	Belongs to the cullin family	Folding, sorting, and degradation	-20.363	3824.598	<0.001	0.043
	50S ribosomal protein L37Ae	Ribosomal component	-20.352	3555.674	<0.001	0.031
	Gar1/Naf1 RNA binding region	Pre-mRNA processing<br>Pseudouridine biosynthesis	-20.352	3555.674	<0.001	0.031
	Catalyzes the CTP-dependent phosphorylation of riboflavin (vitamin B2) to form flavin mononucleotide (FMN)	Archeal riboflavin metabolism	-20.352	3555.674	<0.001	0.031
	COG1413 FOG HEAT repeat	Photosynthesis - antenna proteins	-20.352	3555.674	<0.001	0.031
	PFAM Formate nitrite transporter	Weak acid transporters	-20.352	3555.674	<0.001	0.031
	RNA-binding protein involved in rRNA processing	Ribosome biogenesis<br>Pseudouridine biosynthesis	-20.352	3555.674	<0.001	0.031
	TIGRFAM RNA methyltransferase, TrmH	Transfer RNA biogenesis	-20.352	3555.674	<0.001	0.031
	RNA methyltransferase, TrmH family, group 1	Transfer RNA biogenesis	-20.352	3555.674	<0.001	0.031
	COG3836 2,4-dihydroxyhept-2-ene-1,7-dioHealthic acid aldolase	Tyrosine metabolism	-20.352	3555.674	<0.001	0.031
	Binds to 23S rRNA. One of the proteins that surrounds the polypeptide exit tunnel on the outside of the ribosome	Ribosomal component	-20.352	3555.674	<0.001	0.031
	Belongs to the eukaryotic ribosomal protein eL18 family	Ribosomal component	-20.352	3555.674	<0.001	0.031
	involved in conversion of glycerol to dihydroxy-acetone	Incomplete oxidation of glycerol (Acetobacteria)	-20.268	3516.885	<0.001	0.036
	COG2064 Flp pilus assembly protein TadC	Bacterial secretion system	-20.162	3281.428	<0.001	0.034
	adenosine kinase activity	ATP + adenosine = ADP + AMP	-20.162	3205.851	<0.001	0.031
	TIGRFAM methyltransferase, MtaA CmuA family	Methane metabolism	-19.911	3035.848	<0.001	0.042
	Shwachman-Bodian-Diamond syndrome (SBDS) protein	Poorly characterized	-19.911	2955.685	<0.001	0.037
	FMN biosynthetic process	FMN synthesis	-19.911	3099.002	<0.001	0.046
	PFAM PhoU family protein	Phosphate uptake	-19.911	3028.820	<0.001	0.042
	TIGRFAM Coenzyme F420 hydrogenase, subunit gamma	Methane metabolism	-19.757	2881.500	<0.001	0.046
	Spore germination protein GerE	Sporulation	-19.757	2435.586	<0.001	0.028
	PFAM sodium calcium exchanger membrane region	Ca2+ homeostasis	-19.757	2704.730	<0.001	0.036
	PFAM Shwachman-Bodian-Diamond syndrome	Poorly characterized	-19.757	2840.473	<0.001	0.044
	Located at the polypeptide exit tunnel on the outside of the subunit	Chloroplast component	-19.575	2628.041	<0.001	0.046
	peptidoglycan binding domain	Bacterial cell wall degradation	-19.575	2628.041	<0.001	0.046
	adenosine kinase	ATP + adenosine = ADP + AMP	-19.575	2470.102	<0.001	0.036
	Bacterial toxin homologue of phage lysozyme, C-term	Bacteroicin	-19.575	2430.428	<0.001	0.032
	box H/ACA snoRNA binding	Protein synthesis and mRNA splicing	-19.575	2646.634	<0.001	0.047
	Purine nucleoside phosphorylase DeoD-type	Purine metabolism<br>Pyrimidine metabolism<br>Nicotinate and nicotinamide metabolism	-19.575	2224.849	<0.001	0.028
	Nicotianamine synthase protein	Nicotinate and nicotinamide metabolism	-19.575	2224.849	<0.001	0.028
	Domains LysM, LysM, NLPC_P60	Peptidoglycan synthesis	-19.575	2224.849	<0.001	0.028
	cAMP-dependent protein kinase activity	cAMP-dependent protein kinase	-19.575	2699.328	<0.001	0.049
	SMART Excinuclease ABC C subunit domain protein	Nucleotide excision repair	-19.575	2699.328	<0.001	0.049
	excinuclease ABC, C subunit	cAMP-dependent protein kinase	-19.575	2699.328	<0.001	0.049

***logFC = log fold-change; SE = standard error; FDR = false discovery rate**

Supplementary Table 4. Underrepresented COG/KOG/NOG Orthology genes in MetS and T2DM vs healthy [Continued]

Study Group	COG/KOG/NOG Description	Possible Chemical and Biological Participation	logFC	SE	p-value	FDR
MetS	Excinuclease ABC, C subunit	cAMP-dependent protein kinase	-19.575	2699.328	<0.001	0.049
	CAMP-dependent protein kinase catalytic subunit	cAMP-dependent protein kinase	-19.575	2699.328	<0.001	0.049
	SMART Excinuclease ABC, C subunit	Nucleotide excision repair	-19.575	2699.328	<0.001	0.049
	histone acetyltransferase	Acetylation	-19.450	2414.181	<0.001	0.042
	Dynein assembly factor with WDR repeat domains 1	Signaling and cellular processes	-19.352	2240.894	<0.001	0.037
	PFAM Carbohydrate kinase	Carbohydrate metabolism	-19.352	2322.561	<0.001	0.044
	Aldehyde oxidase and xanthine dehydrogenase, a b hammerhead domain	Branched-chain amino acid degradation<br>Tyrosine metabolism<br>Purine metabolism<br>Tryptophan metabolism<br>Nicotinate and nicotinamide metabolism<br>Vitamin B6 metabolism	-1.941	0.485	<0.001	0.042
	Thiamine pyrophosphate enzyme, C-terminal TPP binding domain protein	Glycolysis / Gluconeogenesis<br>Citrate cycle<br>Branched-chain amino acid degradation<br>Pyruvate metabolism<br>Propanoate metabolism<br>Thiamine metabolism	-1.852	0.475	<0.001	0.049
	Cation diffusion facilitator family transporter	Removal of divalent metal ions	-1.374	0.323	<0.001	0.049
	Signal peptidase	Signaling and cellular processes	-1.164	0.220	<0.001	0.028

***logFC = log fold-change; SE = standard error; FDR = false discovery rate**

Supplementary Table 4. Underrepresented COG/KOG/NOG Orthology genes in MetS and T2DM vs healthy [Continued]

Study Group	Description	Possible Chemical and Biological Participation	logFC	SE	p-value	FDR
T2DM	Protein of unknown function (DUF1566)	Poorly characterized	-20.430	2282.572	<0.001	0.004
	Putative heavy-metal chelation	Poorly characterized	-20.919	3486.243	<0.001	0.008
	isochorismatase hydrolase	Enterobactin biosynthesis	-20.073	2300.492	<0.001	0.008
	CAAX amino terminal protease family	Metalloprotease activity	-20.206	2517.943	<0.001	0.010
	Domains LysM, LysM, NLPC_P60	Peptidoglycan synthesis	-20.736	3651.702	<0.001	0.025
	Catalyzes the condensation of ribulose 5-phosphate with formaldehyde to form 3-hexulose 6-phosphate	Pentose-Phosphate pathway<br>Methane metabolism	-19.736	2250.978	<0.001	0.026
	Psort location CytoplasmicMembrane, score 7.63	Poorly characterized	-20.593	3501.926	<0.001	0.027
	TIGRFAM Bacteroidetes-specific	Poorly characterized	-19.919	2563.390	<0.001	0.031
	COG3836 2,4-dihydroxyhept-2-ene-1,7-dioic acid aldolase	Tyrosine metabolism	-20.513	3539.687	<0.001	0.031
	Branched-chain amino acid ABC transporter, ATP-binding protein	ABC Transporters	-20.513	3539.687	<0.001	0.031
	Bacterial toxin homologue of phage lysozyme, C-term	Bacteroicin	-19.736	2419.557	<0.001	0.031
	Ferredoxin thioredoxin reductase catalytic beta chain	Oxidorreductase activity	-19.736	2419.557	<0.001	0.031
	ferredoxin-thioredoxin reductase activity	Oxidorreductase activity	-19.736	2419.557	<0.001	0.031
	COG2064 Flp pilus assembly protein TadC	Bacterial secretion system	-20.324	3266.698	<0.001	0.031
	SMART Extracellular solute-binding protein, family 3	ABC Transporters	-19.736	2515.696	<0.001	0.040
	PFAM Glycoside hydrolase, family 42	Polysaccharides degradation	-19.513	2286.847	<0.001	0.040
	PFAM Carbohydrate kinase	Carbohydrate metabolism	-19.513	2312.145	<0.001	0.043
	PFAM GumN family protein	Acetobacter uncharacterized protein	-20.612	4029.416	<0.001	0.043
	uroporphyrin-III c-methyltransferase	Porphyrin and chlorophyll metabolism	-20.206	3303.975	<0.001	0.043
	PFAM Bacterial transcription activator, effector binding	Transcription activation	-1.335	0.291	<0.001	0.043
	cAMP-dependent protein kinase activity	cAMP-dependent protein kinase	-19.736	2687.166	<0.001	0.043
	CAMP-dependent protein kinase catalytic subunit	cAMP-dependent protein kinase	-19.736	2687.166	<0.001	0.043
	TIGRFAM tRNA-guanine transglycosylase, various specificities	Transfer RNA biogenesis	-19.736	2689.869	<0.001	0.043
	Pfam:X_fast-SP_rel	Bacterial pathogenesis	-19.736	2705.085	<0.001	0.043
	Prokaryotic acetaldehyde dehydrogenase, dimerisation	Phenylalanine metabolism<br>Benzoate degradation<br>Pyruvate metabolism<br>Dioxin degradation<br>Xylene degradation<br>Butanoate metabolism	-20.073	3221.556	<0.001	0.043
	phage regulatory protein, rha family	Phage replication	-1.369	0.307	<0.001	0.044
	TipAS antibiotic-recognition domain protein	Multidrug resistance regulator	-20.430	3907.343	<0.001	0.046
	regulatory protein, LuxR	Transcription regulation	-19.525	2497.466	<0.001	0.047
	PFAM Sporulation	Sporulation	-1.656	0.394	<0.001	0.047
	Alpha-n-arabinofuranosidase a	Amino sugar and nucleotide sugar metabolism	-19.513	2512.773	<0.001	0.047

***logFC = log fold-change; SE = standard error; FDR = false discovery rate**

Supplementary Table 4. Underrepresented COG/KOG/NOG Orthology genes in MetS and T2DM vs healthy [Continued]

Study Group	Description	Possible Chemical and Biological Participation	logFC	SE	p-value	FDR
T2DM	Acetyl-CoA acetyltransferase of cellular organisms UniRef RepID THIL_CUPNH	Fatty acid degradation<br>Synthesis and degradation of ketone bodies<br>BCAA degradation<br>Lysine degradation<br>Benzoate degradation<br>Tryptophan metabolism<br>Pyruvate metabolism<br>Glyoxylate and dicarboxylate metabolism<br>Propanoate metabolism<br>Butanoate metabolism	-19.736	2814.015	<0.001	0.047
	PFAM ErfK YbiS YcfS YnhG family protein	Peptidoglycan biosynthesis	-1.360	0.313	<0.001	0.047
	Copper/zinc superoxide dismutase (SODC)	Superoxide radicals removal	-1.001	0.221	<0.001	0.047
	Golgi vesicle prefusion complex stabilization	Golgi vesicle complex	-19.513	2539.006	<0.001	0.047
	C4-dicarboxylate ABC transporter	Two-component system	-19.736	2840.028	<0.001	0.047
	Putative modulator of DNA gyrase	DNA replication	-19.513	2570.514	<0.001	0.048
	Plays an important role in the de novo pathway and in the salvage pathway of purine nucleotide biosynthesis. Catalyzes the first commited step in the biosynthesis of AMP from IMP	Purine metabolism	-20.073	3400.890	<0.001	0.048
	DNA repair	DNA recombination and repair	-19.919	3166.619	<0.001	0.048
	N-acetylglucosamine-1-phosphodiester alpha-N-acetylglucosaminidase	Lysosome	-20.073	3431.513	<0.001	0.049

***logFC = log fold-change; SE = standard error; FDR = false discovery rate**

Supplementary Table 5. KEGG significant pathways in MetS and T2DM vs healthy

Study Group	Description	logFC	SE	p-value	FDR
MetS + T2DM	Nitrogen metabolism	0.072	0.014	<0.001	0.006
	Nonribosomal peptide structures	-0.204	0.046	<0.001	0.036
	Melanogenesis	-0.625	0.148	<0.001	0.041

***logFC = log fold-change; SE = standard error; FDR = false discovery rate**

Supplementary Table 6. KEGG significant pathways in MetS vs T2DM

Study Group	Description	logFC	SE	p-value	FDR
MetS vs T2DM	Nitrogen metabolism	0.039	0.008	<0.001	0.011
	Mineral absorption	-0.106	0.023	<0.001	0.018

***logFC = log fold-change; SE = standard error; FDR = false discovery rate**
